# Characterization and Comparison of the Tissue-Related Modules in Human and Mouse

**DOI:** 10.1371/journal.pone.0011730

**Published:** 2010-07-22

**Authors:** Ruolin Yang, Bing Su

**Affiliations:** 1 State Key Laboratory of Genetic Resources and Evolution, Kunming Institute of Zoology, Chinese Academy of Sciences, Kunming, China; 2 Kunming Primate Research Center, Chinese Academy of Sciences, Kunming, China; 3 Graduate School of the Chinese Academy of Sciences, Beijing, China; BC Centre for Excellence in HIV/AIDS, Canada

## Abstract

**Background:**

Due to the advances of high throughput technology and data-collection approaches, we are now in an unprecedented position to understand the evolution of organisms. Great efforts have characterized many individual genes responsible for the interspecies divergence, yet little is known about the genome-wide divergence at a higher level. Modules, serving as the building blocks and operational units of biological systems, provide more information than individual genes. Hence, the comparative analysis between species at the module level would shed more light on the mechanisms underlying the evolution of organisms than the traditional comparative genomics approaches.

**Results:**

We systematically identified the tissue-related modules using the iterative signature algorithm (ISA), and we detected 52 and 65 modules in the human and mouse genomes, respectively. The gene expression patterns indicate that all of these predicted modules have a high possibility of serving as real biological modules. In addition, we defined a novel quantity, “total constraint intensity,” a proxy of multiple constraints (of co-regulated genes and tissues where the co-regulation occurs) on the evolution of genes in module context. We demonstrate that the evolutionary rate of a gene is negatively correlated with its total constraint intensity. Furthermore, there are modules coding the same essential biological processes, while their gene contents have diverged extensively between human and mouse.

**Conclusions:**

Our results suggest that unlike the composition of module, which exhibits a great difference between human and mouse, the functional organization of the corresponding modules may evolve in a more conservative manner. Most importantly, our findings imply that similar biological processes can be carried out by different sets of genes from human and mouse, therefore, the functional data of individual genes from mouse may not apply to human in certain occasions.

## Introduction

How phenotypes are determined by genotypes is of fundamental importance for understanding the principles underlying the evolution of organisms. Many insights have been gained by the traditional comparative genomics approaches which often compare the between-species difference at the sequence level [1], [2]. So far, researchers have identified a large body of conserved [3], [4] or rapidly evolving [5], [6] protein-coding regions and cis-regulatory elements, which are either involved in essential biological activities across multi-organisms or contributing to species-specific phenotypes.

Thanks to the recent advances of high-throughput techniques, a variety of biological data (including whole-genome expression profile, protein-protein interaction, genetic interaction, DNA-protein binding data etc.) are accumulating at a rapid pace in data repositories, providing an invaluable resource from which data-driven hypotheses have been proposed. The large-scale gene expression profiles are especially useful for exploiting cell behavior since they record the genome-wide tempo-spatial dynamics of genes. Comparing the expression pattern between related species [7], [8] or among multiple organisms [9] provides an alternative approach to investigate the inter-species divergence. In recent years, some advanced methods have been developed to cope with the large-scale gene expression data. For example, Segal *et al.* [10] introduced a probabilistic method to identify modules from gene expression data, which not only identifies the co-regulated genes and the condition under which regulation occurs, but also their regulators. Zhang and Horvath [11] proposed a weighted gene coexpression network analysis (WGCNA) method which can define modules according to a “weighted” topological overlap measurement, a variant of topological overlap originally proposed by Ravasz *et al* [12].

As one of the model organisms, mouse provides pivotal and rich materials for understanding the biology of human, particularly in the biopharmaceutical field. However, some fundamental problems such as how much evolutionary divergence separates human from mouse and to what extent the experimental observations on mouse can be applied to human are still poorly understood. A few studies have attempted to investigate these problems. For instance, Tsaparas *et al.* [13] compared the genomic divergence of gene expression between human and mouse by resolving the expression profiles into species-specific coexpression networks. They revealed that despite essentially identical at the global level, the human and mouse coexpression networks are highly divergent at the local level. Odom *et al.* [14] also demonstrated that the binding sites for highly conserved transcriptional factors have diverged extremely between human and mouse by mapping the binding of four representative transcriptional factors to 4,000 human-mouse orthologs.

The concept of module has been widely used in literatures; however, its definition is relatively vague. The traditional clustering approaches, such as K-means clustering [15], self-organizing maps [16] and hierarchical clustering [17] often associate a cluster of genes with a module, in which all the involved genes display similar expression dynamics across predefined conditions. Based on the idea that a group of genes can only be co-regulated and function in certain conditions, e.g. under environmental change, the stimuli of specific agents, special developmental phase and specific tissues/organs, Ihmels *et al.* [18] proposed a novel algorithm (signature algorithm) to detect the modules from the gene expression profiles. They termed such a combined group of genes and conditions that trigger the co-regulation of the associated genes as a “transcription module”. Ihmels *et al.* devised the iterative signature algorithm (ISA), (an improved version of the signature algorithm) that has more rigorous mathematics and can capture the hierarchical structure of modules [19].

In order to achieve a deeper understanding of the evolutionary divergence between human and mouse in a higher order, we compiled two gene expression matrixes, which included 6,200 pairs of one-to-one orthologs across 29 homologous tissues for human and mouse. Inspired by the work of Ihmels and colleagues, we identified the tissue-related modules in the two species using the iterative signature algorithm (ISA) [20], and we characterized these modules and compared the genomic divergence of human and mouse in the context of modules.

## Results and Discussion

Before we began to identify the modules, we examined the distribution patterns of gene expression values. As shown in Figure S1, despite of the consistently higher expression level (signal intensity) in human than that in mouse (which is likely caused by the different normalization processes or other factors), on the whole, the gene expression patterns across the tissues are similar within each species and the trends are also similar between species regardless of their different absolute expression levels. The overall expression difference between human and mouse does not create significant bias in our analysis because, firstly, the strategy of our module analysis was a two-step processes, first identifying modules in each species using ISA and then comparing the modules of human and mouse; Secondly, the two datasets were profiled by an united microarray platform of the same lab, therefore, the two raw gene expression data (6200 genes×29 tissues) should be comparable.

The modules identified by ISA are threshold-dependent. Given that the number of the tissues (29) is much less than that of the genes (6,200) in the expression data, we first evaluated the performance of module discovery by adjusting the condition threshold (Tc = 1.0, 1.25, 1.5, 1.75 and 2.0), while the gene threshold (Tg) was fixed at a somewhat arbitrary value, 3.0. The results showed that the number of the refined post-merged modules (RMP modules) in both species was maximized when Tc was 1.5. We then refined the gene threshold while keeping Tc = 1.5. Accordingly, we identified the maximal number of modules under Tc = 1.5 and Tg = 3.0 (Figure S2), and we took into account the following factors: 1) much more unrelated genes might randomly wind up into modules simply due to noise under non-stringent parameters; 2) the maximal number of modules is more powerful for the statistical analysis of “evolutionary pattern” (because the analysis is based on the module context); 3) we believe that it would better represent the overlapped structure of modules under current thresholds; and 4) crucially, the module number determined by alternative criterion are limited. All the other analyses presented below were based on the RMP modules identified by applying Tc = 1.5 and Tg = 3.0 (Actually, the modules identified using other parameters showed similar results regarding the interspecies differences, but these modules were not suitable for the analysis of evolutionary pattern due to their limited number).

### The contents of modules diverge greatly between human and mouse

**Figure 1:**
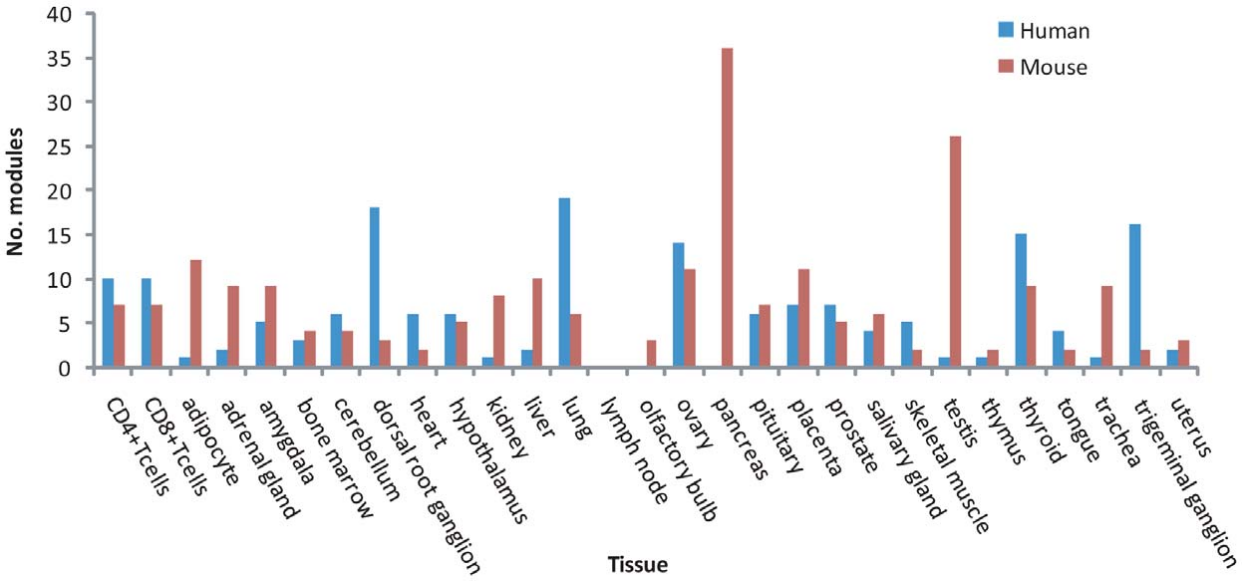
Uneven distribution of the modules in the 29 tissues. The distribution pattern of modules diverges extensively between human and mouse. For instance, in the pancreas, adipocyte, kidney, testis and so on, there are much more modules identified in mouse than that in human, whereas the opposite observation is seen in the dorsal root ganglion, lung and trigeminal ganglion etc.

Totally, from the two expression data including 6,200 pairs of one-to-one orthologs, we identified 52 and 65 tissue-related modules (Table S1 and S2) containing 509 and 528 genes in human and mouse, respectively, among which 148 pairs of orthologs are shared between species. The number of genes in a human (mouse) module ranges from 11(10) to 58(63). On average, a module is comprised of 29.5 genes associated with 3.3 tissues in human and 27.3 genes associated with 3.4 tissues in mouse. However, these modules are unevenly distributed in the 29 tissues. Also, the distribution pattern of modules in the two species diverges dramatically (see Figure 1). For example, the lung has the largest number of modules in human, while in mouse it is the case for the pancreas. There are only one or two modules identified in the thymus in both species. No module was detected in the lymph node in the two species and pancreas-associated modules were discovered only in mouse, which is likely caused by the following reasons: 1) the modules identified by ISA are threshold-dependent, hence, it is possible that the current threshold is too strict to identify a module in these tissues; 2) Sampling bias may have uncertain effect on module's identification simply due to the biased expression of the 6,200 genes in different tissues; 3) we may occasionally leave out some modules because the search space is too large (given that 6,200 genes) though we have intended to identify all the modules exhaustively; 4) as will be shown below, the contents of the between-species modules have diverged greatly, hence, we often cannot identify the mouse module even when we input a human module (a list of human orthologs) to ISA, and vice versa.

In a previous study, Su *et al.* [21] have investigated the effect of chromosomal organization on the expression mode of genes and determined hundreds of RCTs (chromosomal regions of correlated transcription). They observed that RCTs harboring genes highly expressed in the olfactory bulb presented in mouse but not in human, and attributed it to different physiology between the two species. We observed the similar pattern in the olfactory bulb-expressed modules.

In comparison with traditional clustering methods, the modules identified by ISA are associated with conditions. For our modules, they are combinations of a group of genes and tissues where the co-regulation occurs. We found that a variety of tissues often share the same modules. For example, there are two mouse modules (module 28, 29 in Table S2) co-regulated in kidney and liver, which is consistent with a previous study by Freeman *et al.* [22], who observed that these organs are near to or even connected in a graphic transcriptional networks in terms of clusters (a group of inter-connected genes).

**Figure 2:**
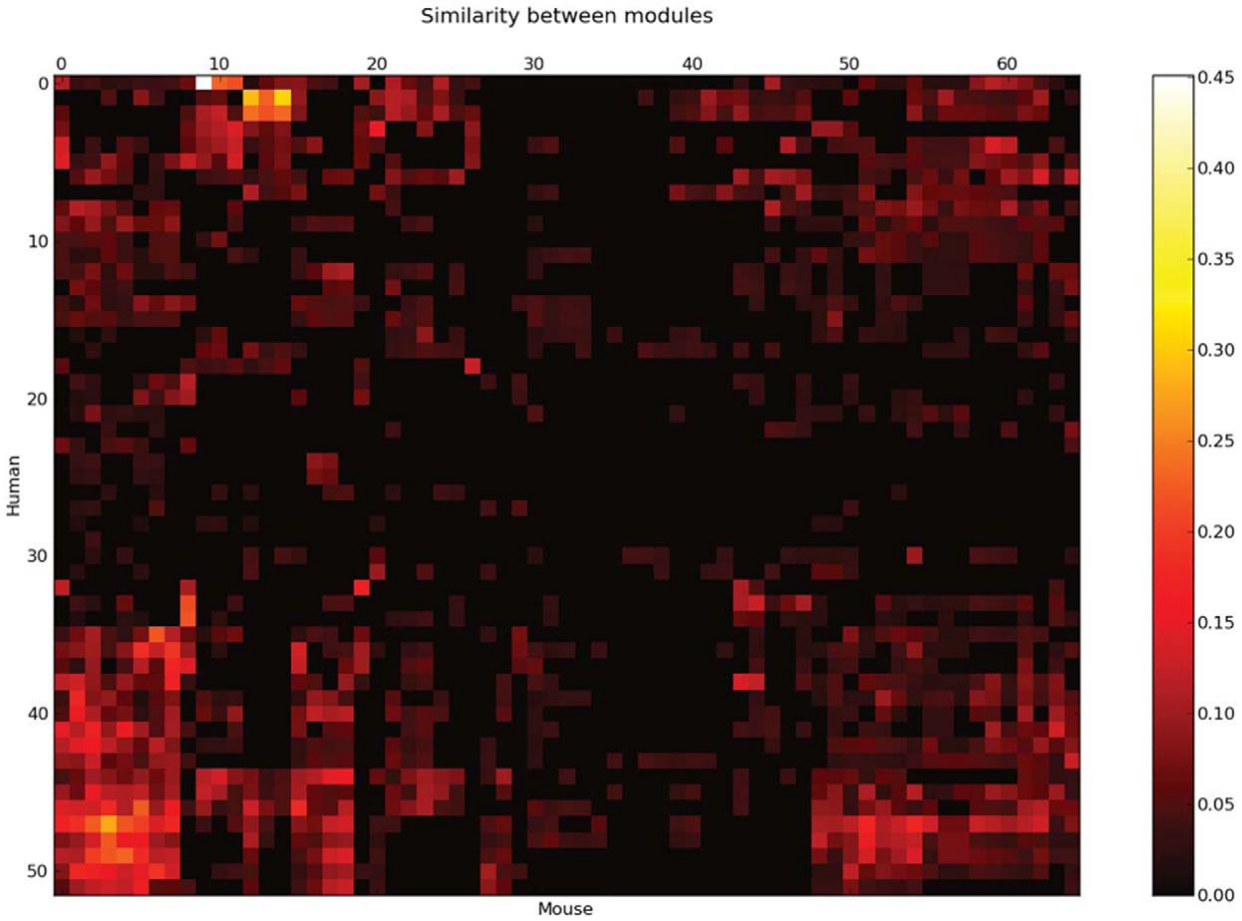
All-to-all comparison of modules between human and mouse. The heat map (bi-clustered) displays a globally low similarity between the inter-species modules. The similarity between a pair of module is calculated by Eqs. (**1**).

In order to further examine the difference of modules between human and mouse, we compared the pair-wise modules derived from human and mouse with the use of similarity measurement calculated by Eqa. **(1)** (see Methods). As shown in Figure 2 and Figure S3, we can hardly find any pairs of modules with high similarity between species. Meanwhile, in order to further explore the relationship of modules between the two species, we conducted a hierarchical clustering of all the modules (Figure S4). The dendrograph indicated that all of the modules were separated into two “biggest” clusters, one harboring modules, the overwhelming majority of which are human-derived, and the other containing all but one mouse-derived modules. Taken together, the results suggested that the composition of the modules diverged extensively between the two species.

**Figure 3:**
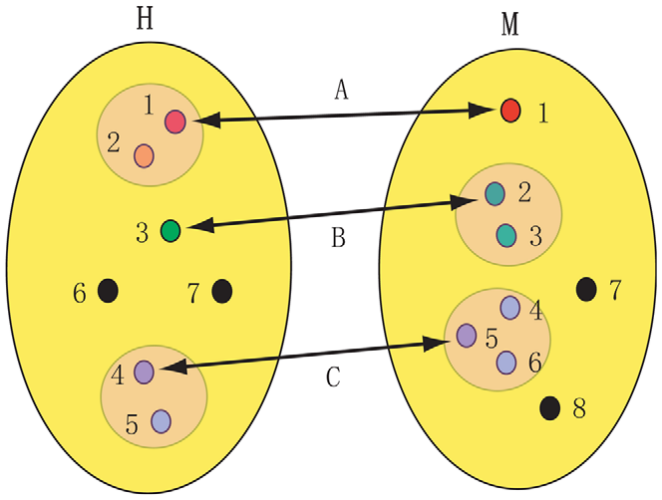
Schematic illustration of the modified similarity. The original similarity between modules H and M is 3/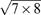 = 0.401; while the modified similarity, which integrates with the paralog information, is equal to ((5+6)/2)/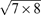 = 0.735. The two big yellow ovals denote two modules from human and mouse, respectively. The four middle cycles highlight the paralogous relationship. Small cycles denote genes and the arrows link the orthologs.

**Figure 4:**
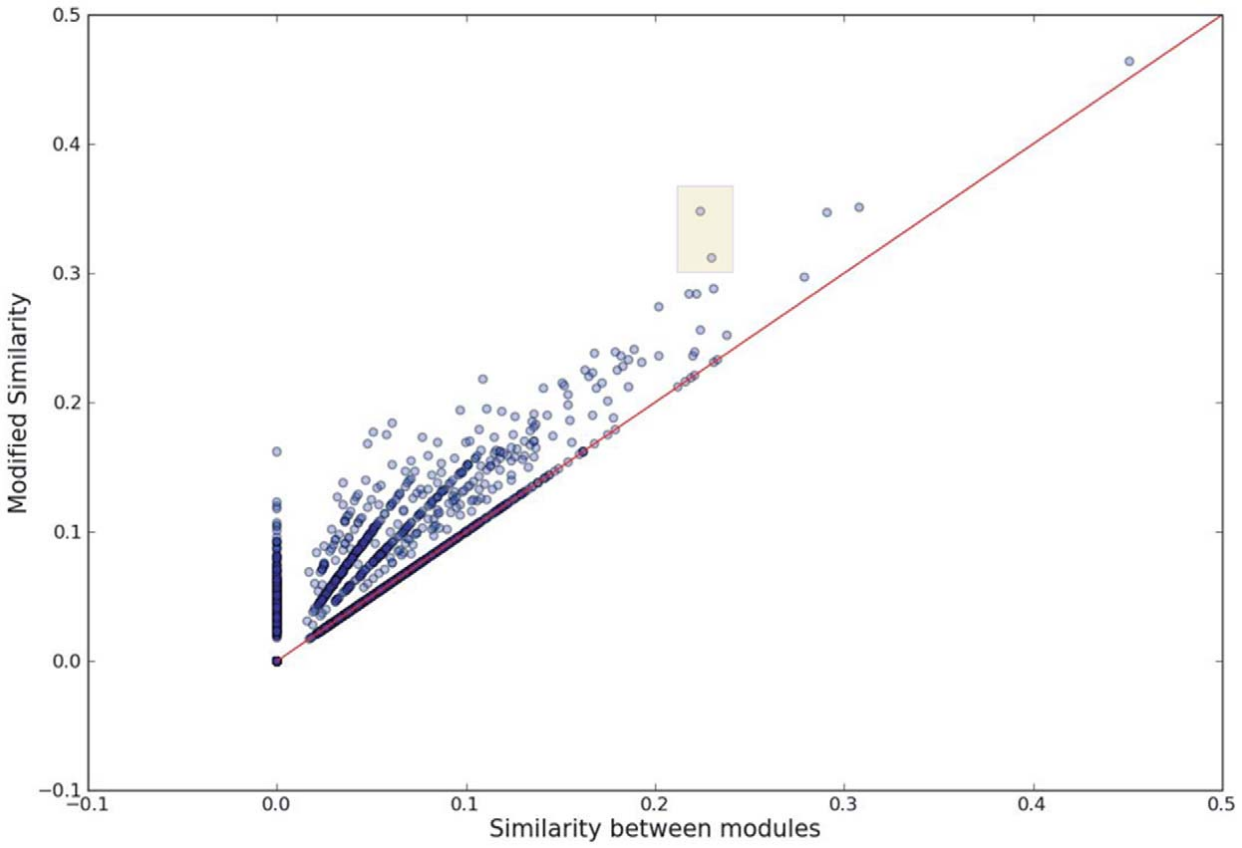
Comparison between the original and the modified module similarity. The scatter plot (including 52
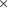
65 points) displays a more or less increase for most of the original similarities; while, on the whole, few inter-species module pairs have a relatively high modified similarity. The similarity of the two points highlighted in the shadow rectangle displays a relatively big boost.

**Figure 5:**
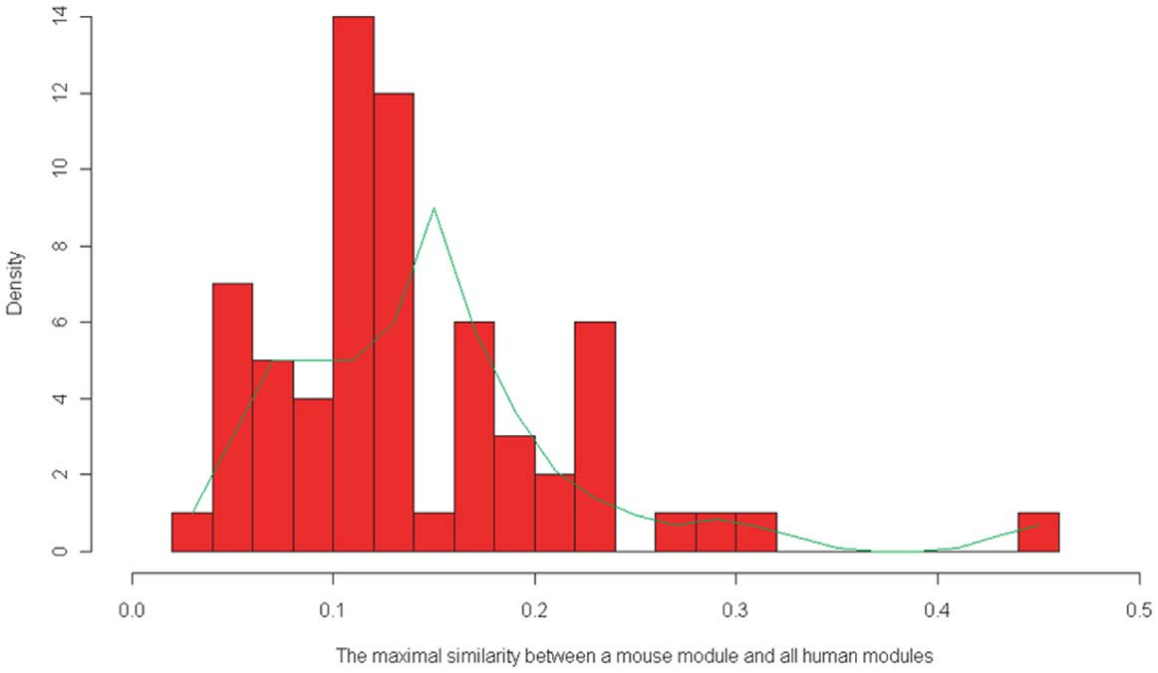
Histograph of the maximal similarity for the 65 mouse modules to all the human modules. The trend line is fitted by the lowess algorithm [54]. This plot displays a few pairs of human-mouse modules with relatively high similarity.

Considering that genes often “group” into gene sets and provide mutual functional backups resulting from genetic redundancy [23], [24], we further investigated the modified similarity for each pair of modules (one from human and the other from mouse) by taking into account the paralogs (see Figure 3). We observe that there is an increase for most of the original similarities, but the majority of the modified similarities are still less than 0.3 (see Figure 4). We then ask whether there are a few “conserved” modules among these modules. For each mouse module M, we define its counterpart which has the maximal similarity to M in human. As illustrated in Figure 5, the histograph of the maximal similarity showed that more than half of the pairs share less than 15% genes, and there are only four pairs of modules with relatively high between-species similarity. For instance, the first pair of modules, which are specifically expressed in the liver, have 45% similarity. The second pair showed ∼28% similarity, both of which are highly expressed in the lung, but highly suppressed in the CD4+ and CD8+ T cell lines. Interestingly, the remaining two pairs are composed of a human module associated with the amygdale, cerebellum and hypothalamus, and two mouse counterparts, which are either highly expressed in the amygdale, cerebellum, hypothalamus, dorsal root ganglion and olfactory bulb, or dominant in the dorsal root ganglion and trigeminal ganglion. It is possible that the two mouse counterparts may originate from one de facto module, which was artificially split into two in the subsequent module-merging process because the similarity between them is high(0.558).

Furthermore, in order to evaluate the significance of the maximal similarity shown in Figure 5, we conducted a simulation analysis according to the following rules: 1) we produced a similarity matrix which was shown in Figure 2, with its row corresponding to 65 mouse modules, and the column corresponding to 52 “simulated” human modules, all of which were sampled from the 509 module-associated human genes, while keeping the number of genes per “simulated” module the same as the real human data; 2) for every mouse module, we determined the maximal similarity by virtue of the 52 “simulated” modules as mentioned above; 3) We repeated 1) and 2) 1,000 times, and got 65,000 values totally, Our data showed that only less than one-third of the maximal similarity has value larger than the 95% quantile of the simulated dataset (Figure S5). Together, the results presented suggest that the genome of human and mouse have diverged dramatically at the module level, which is consisted with a previous study [13] reporting that only less than 10% of co-expressed gene pair relationships are conserved between human and mouse.

### High expression coherence of the modules

**Figure 6:**
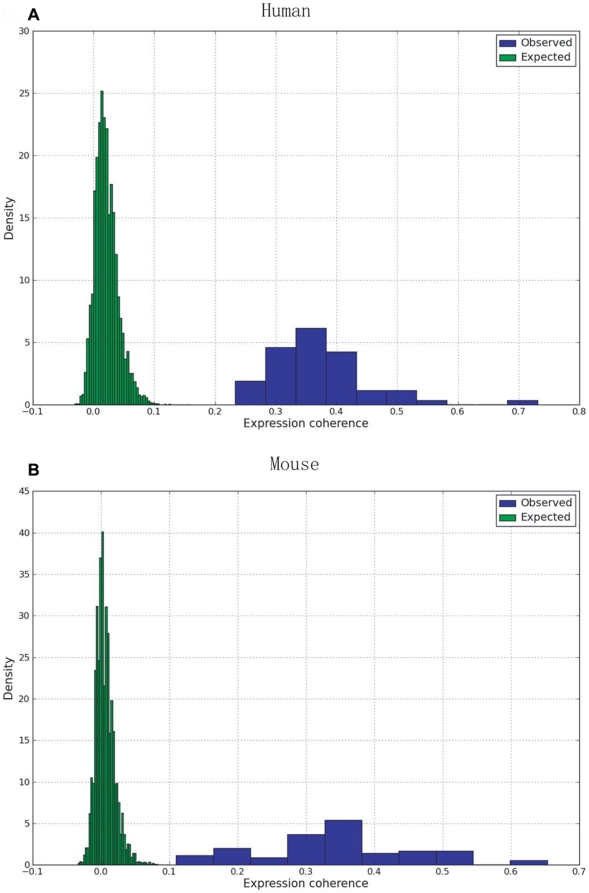
Expression coherence of modules compared with that of their random expectations. Both in human (A) and mouse (B), all the modules identified here represent significant higher expression coherence than the random expectations. The minimal z-score for the mouse modules is up to 7.27.

Functionally related genes are often co-expressed [25], [26] and co-regulated genes also tend to frequently interact with each other [27]. To identify potentially functional associations with a group of predefined genes, Pujana *et al.* [28] proposed the method of assembling candidate genes which are highly co-regulated with these target genes. As a proxy of expression coherence, the averaged Pearson correlation coefficient (PCC) was evaluated for each module. Following Wang and Zhang [29], we used z-score to measure the deviation of the expression coherence of a module from its random expectation. The results indicate that all the identified modules have significant high expression coherence, compared with the controls (see Figure 6). For example, the minimal z-score is 10.90 for the human modules, and 7.27 for the mouse modules. Since the principle of ISA differs from the traditional approaches, such as the hierarchical clustering method [17], which group genes by taking into account the correlation information measured over all conditions, the prevalent high expression coherence of the modules identified herein suggests that these modules have a high probability of acting as the tightly-related functional entities.

### Evolutionary pattern of genes in the module context

The basic activities in a cell are well conceptualized as a complex network, where the immense genes and their products interplay to execute different functions sequentially. Accordingly, the evolutionary pattern of each gene may be restricted by its “niche”, the neighbor genes which directly interact with the gene and the conditions where the gene expresses.

**Figure 7:**
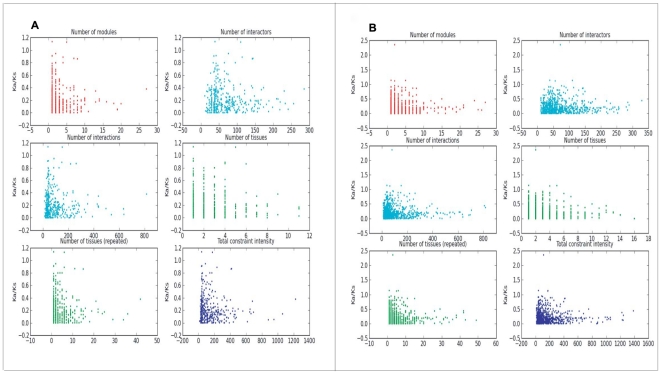
Relationship between the evolutionary rate of a gene and its six characteristic quantities. The scatter plot shows that the evolutionary rate of genes is negatively correlated with the corresponding “total constraint intensity” both for (A) human and (B) mouse.

We sought to investigate the relationship between the evolutionary rate of a gene, *i.e.* the ratio of the rate of non-synonymous substitutions (Ka) versus the rate of synonymous substitutions (Ks), and its six characteristic quantities specified in a framework of module context (see Materials and Methods). The scatter plots (Figure 7) show that all these variables appear to be negatively correlated with the evolutionary rate. Table 1 summarizes the results with respect to correlation coefficients and the corresponding P-values. Strikingly, the evolutionary rate of a gene is negatively correlated (despite weakly) with its “total constraint intensity”, which is defined in the module context as a proxy of multiple constraints (of co-regulated genes and tissues where the co-regulation occurs) on the evolution of genes. (Spearman's ρ = −0.086, P = 0.013 for human; ρ = −0.066, P = 0.049 for mouse).

**Table 1:** Relationship between the evolutionary rate and the characteristic quantities.

	Pearson's r		P-value		Spearman's ρ		P-value	
	Human	Mouse	Human	Mouse	Human	Mouse	Human	Mouse
**#modules**	−0.084	−0.036	0.101	0.470	−0.089	−0.031	0.018	0.404
**#interactors**	−0.060	−0.042	0.238	0.400	−0.034	−0.022	0.332	0.518
**#interactions**	−0.075	−0.046	0.144	0.359	−0.039	−0.031	0.255	0.357
**#tissues**	−0.149	−0.110	0.004	0.027	−0.124	−0.097	0.001	0.007
**#tissues (repeated)**	−0.104	−0.054	0.041	0.276	−0.120	−0.075	0.001	0.033
**Total constraint intensity**	−0.101	−0.067	0.049	0.183	−0.086	−0.066	0.013	0.049

#: number of; 384 human and 404 mouse genes were counted, respectively.

We further dissected the “total constraint intensity” of a gene into two components, the condition complexity, for which we refer to the number of environments (tissues) where the whole modules of the gene actively expressed, and the scale of the neighbor genes. We then examined their association with the evolutionary rate. Our results show that the condition complexity of a gene—whether calculated as “Number of tissues” or “Number of tissues (repeated)”—is significantly negatively correlated with its evolutionary rate (see Figure 7 and Table 1), which is consistent with a previous study [7]. Additionally, the previous study established an association between the evolutionary rate of genes and the breadth of expression, i.e. the number of tissues in which a gene is expressed. Our data proposes a reasonable explanation that the evolutionary constraint on genes by tissues may act through the associated modules.

Simultaneously, we investigated the relationship between the evolutionary rate of a gene and the number of its neighbor genes. Contrary to our expectation, all the correlations are not significant, though they display a week negative correlation. In 2002, Fraser *et al.* [30] pioneered a study reporting that the proteins with more interactors evolve more slowly. Fraser extended the study in view of the modularity, and revealed that the intra-module hub genes evolve more slowly than the inter-module ones in a yeast protein-protein interactome [31]. Considering that: 1) the interplay between genes and/or their products is mediated, either by direct physical interaction, or through indirect regulatory processes; 2) widespread modular epistasis among genes may serve as a common principle underpinning the genetic robustness of genomes (Segre *et al.* [32] discovered that modular epistasis between genes is pervasive in the yeast metabolism), we speculate that the correlation between the evolutionary rate of genes and their corresponding “Number of interactors” and “Number of interactions” which we defined in the context of transcriptional module could be stronger and more significant than what have been revealed in the previous studies. However, we did not observe the preconceived results. There are three possible reasons partially accounting for the observations: 1) Modules are organized in a hierarchical manner. The higher thresholds were applied in the ISA, the tighter modules would be identified [9]. In this study, in order to assemble more modules, we compromised the stringency of modules by adjusting the condition threshold to a small value, 1.5. Theoretically, some of the modules identified may either contain unrelated genes, or be a union of two or more de facto modules, both of which may vitiate our results; 2) The sampling bias, which has been frequently addressed in most of the physical interaction networks [33], has undesirable effect on results. It is also possible that the expression of the 6200 genes has tissue sampling bias, leading to more modules identified in some of the tissues. 3) Other factors, such as the pathway position [34], gene compactness and gene essentiality [35] and the percentage of disordered residues of a protein [36] may have influences on the evolutionary rate of the corresponding gene, which implicitly complicated the relationship between the scale of neighbor genes and the evolutionary pattern.

To address the concerns regarding saturation of evolutionary rate, we first examined the distribution of synonymous substitution rate per synonymous site along the human or mouse lineage. The results showed that all the Ks values, both for human and mouse, are less than one. (Figure S6). Then even after we removed 50 genes with the largest Ks value, the relationship between the evolutionary rate and the characteristic quantities remains the same (data not shown).

In summary, the obvious association between the evolutionary rate of genes and the “total constraint intensity” highlights a possible scenario that the evolutionary constraint on genes may also act at the module level.

### Functional analysis of modules

For each of these modules, we evaluated the functional enrichment using the human or mouse gene ontology (GO) categories for biological processes and molecular functions (see Methods). Setting the cutoff of the corrected P-value at 0.05 and using the default, we detected 47(42) and 52(37) modules which are enriched with at least one GO category in terms of the biological processes (molecular functions) in human and mouse, respectively. Given that the background distribution of the GO terms in our gene lists may differ from the default used by GO Term Finder package [37], we reappraised the functional enrichment and found 36 human and 42 mouse modules indicative of functional enrichment in terms of the biological processes. Overall, the results indicated that most of the modules are organized into functional units.

Considering that the inter-species modules differ extensively in their composition, we next ask whether these seemingly distinct modules still code some common or even essential biological processes in the genomes of human and mouse. First, we compared five pairs of inter-species modules, each of which displays a relatively high overlap. Table S3 lists some basic information of these modules and the overlapped GO enrichment terms between the corresponding modules. We can see that each pair of modules shared several GO terms except for the last pair of modules for which we did not detect overrepresented GO terms in the corresponding module of mouse. Interestingly, the functional overlap (GO annotation: regulation of muscle contraction) emerges in a pair of modules, one of which is highly expressed in the heart and lung in human, while the other is actively expressed in the skeletal muscle, tongue and trachea in mouse. Then we compared the enriched GO terms in most of the homologous tissues except for the lympy node, olfactory bulb and pancreas. We combined all the over-represented GO terms (corresponding to a module) pertaining to certain tissue and counted the overlapped terms between each pair of homologous tissues. The results showed that all the homologous tissues used for the comparison but the pituitary hold at least one common GO term with regard to the biological processes. For example, in testis, the enriched genes in GO annotation are related to male gamete generation and spermatogenesis both in human and mouse; and the adrenal gland has significantly more genes related to the C21-steroid hormone metabolic process and lipid metabolic process than the random expectation. Additionally, the genes associated with anatomical structure development, inflammatory response, multicellular organismal development and response to external stimulus etc. are over-represented in the placenta.

Overall, our results implied that unlike the composition of module which exhibited a great divergence between the human and mouse genomes, the functional organization of the modules may evolve in a more conservative manner.

### Robustness of modules

**Figure 8:**
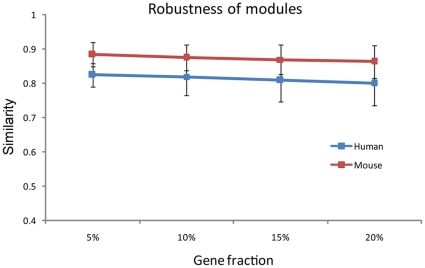
Sensitivity of the modules with respect to the size of the dataset. Shown in the plot are the mean and standard deviation of the similarity between the modules identified when a fraction of data is removed from the raw dataset and those identified with the full dataset.

To address the concerns regarding the robustness of modules, we conducted a sensitivity test by leaving out 5%, 10%, 15% and 20% of the genes from the raw data. Our results demonstrated that the modules are robust. For example, even though we removed up to 20% of the data of the human and mouse expression matrixes, we can still recover modules with a mean similarity of 0.80, and 0.86 to those identified by using the full dataset, respectively (see Figure 8).

### Concluding remarks

Here we systematically identified and characterized the tissue-related modules of human and mouse using the ISA. All these identified modules showed a significant high co-regulation, suggesting a high possibility for them serving as real biological modules. In addition, we investigated the relationship between the evolutionary rate and the characteristic quantities defined in a module context. Our results showed that the evolutionary rate of a gene is significantly negatively related to its “total constraint intensity”, which was defined as a proxy of multiple constraints on the evolution of genes in a module context, whereas the weak negative correlation between the “number of interactors”, “number of interactions” and the corresponding Ka/Ks ratios is not significant. We believe that the availability of more genome-wide measurements of the gene expression profiles across tissues will allow researchers to gain more insights into the evolutionary pattern of genes in the context of modules.

Exemplified by human and mouse, we demonstrated that the inter-species modules may code some common or essential biological processes, despite a relatively big difference between their contents. Remarkably, according to the transcriptional program of mouse hepatocytes carrying human chromosome 21, Wilson *et al.* [38] have recently unraveled that the regulatory sequences between human and mouse have greatly diverged. In a previous study [39], we have found that rhesus macaque performs much better than mouse as an outgroup in identifying human-specific selection, suggesting that a relatively large genetic differences exist between human and mouse. Consistent with these results, our findings have implications on the use of mouse as a model when studying the biology of human, reminding that we should be more cautious of applying the functional data from mouse because the same biological processes in different organisms may be carried out by a group of different genes.

## Materials and Methods

### Gene Expression Data

We downloaded the human and mouse gene expression datasets from GNF Genome Informatics Applications & Data sets (http://wombat.gnf.org) [21]. These datasets cover 79 human and 61 mouse tissues, among which 29 tissues (adipocyte, adrenal gland, amygdala, bone marrow, cerebellum, dorsal root ganglion, heart, hypothalamus, kidney, liver, lung, lymph node, olfactory bulb, ovary, pancreas, CD4+Tcells, CD8+Tcells, pituitary, placenta, prostate, salivary gland, skeletal muscle, testis, thymus, thyroid, tongue, trachea, trigeminal ganglion and uterus) are shared in the two datasets and they are used as homologous tissues for subsequent inter-species comparison. Independent studies have reported that the MAS5-based [40] (an algorithm computing the gene expression values from probe set intensity values) and GC-RMA-based [41] (GC content–adjusted robust multi-array algorithm) gene expression level gave rise to similar results [42], [43], hence, we used the signal intensity (*S*) computed from MAS 5.0 algorithm (MAS5) as gene expression level detected by each probe set. The *S* values were averaged among replicates before analysis. A series of processes were carried out to filter out sub-optimal probe sets (including probe sets that target multiple genes and those whose target gene has multiple probe sets). After that, we screened out 6,200 one-to-one orthologs (and corresponding probe sets) according to the human-mouse orthologs map information downloaded from the Ensembl database (http://www.ensembl.org/). Eventually, we generated a pair of gene expression matrixes (6200 genes 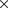
29 tissues) in which the same row and column represent the human-mouse orthologs and homologous tissues, respectively.

### Identification of modules

All the tissue-related modules were identified using the ISA algorithm proposed by Bergmann *et al.* [20] which over-performs many traditional clustering approaches in two main aspects: 1) the modules identified by ISA are highly self-consistent; 2) the genes within a module are allowed to be involved in alternative modules [19]. We determined the modules of the two species, using an exhaustive searching strategy in which a group of genes (the number of these genes ranging from 20 to 50) sampled from the 6,200 orthologs were used as the input gene set both for human and mouse in each round of run of ISA.

### Mergence and refinement of modules

We denoted a module as M (G, T), where G and T are the gene and tissue set of the corresponding module M, respectively. The module similarity between M_i_ (G_i_, T_i_) and M_j_ (G_j_, T_j_) was defined at three levels as:
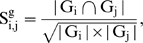
(1)

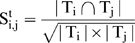
(2)and
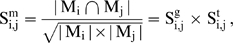
(3)where |…| refers to the size of a set and 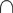 denotes intersection.

We proposed an iterative graph-based module-merging approach (IGMM) to merge a group of modules. The similarity among modules is described as a module relationship graph (MRG) in which the nodes signify modules and an edge links two nodes if their corresponding modules have a similarity above a predefined threshold (for example, all the results in the main text are based on 0.7). The IGMM method is simply stated as follows:

1. All of the pair-wise module similarity between module i and j included in an initial module set MS^0^ = {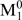,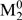,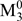,…} are measured according to 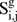;
2. We searched all of the cliques from the MRG, which are defined as fully connected subgraphs of a graph mathematically [44].
3. For each clique, the corresponding modules are coalesce to form a single united module in which genes and tissues remain if they are involved in no less than 80% members of the pre-merged modules.
4. Through the above-mentioned steps, the module set is updated from MS^k−1^ to MS^k^ = {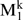,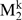,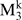,…}. Repeat from step 1 until convergence: MS^k−1^ = MS^k^.

To strictly meet the requirement of consistency, we further refined the post-merged modules. We first computed the similarity between the post-merged modules and its ISA-outputted counterparts using equations (4–6).
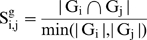
(4)

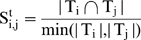
(5)

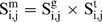
(6)It is worth noting that the formula of module similarity differs from Eqs. (**1** **–** **3**) in which the denominator of Eqs. (**1** **–** **3**) is reformatted as the minimal cardinality of the two sets in Eqs. (**4** **–** **6**). We then selected those post-merged modules which have 100% similarity measured by Eqs. (**4** **–** **6**), when compared with their ISA-outputted counterparts.

### Expression coherence

The module expression coherence is defined as the average of Pearson correlation coefficients of all pair-wise gene expression profiles pertaining to the corresponding module across the 29 common tissues. The statistical significance is assessed by 10,000 independent gene sets randomly sampled from the 6,200 orthologs. To cover the different sizes of these modules, we constructed five controls, four of which are composed of the gene sets with an invariable size, the number of genes in each gene set in the four controls ranging from 20 to 50 in ascending order; while the fifth control consisted of the gene sets with variable size from 20 to 50 which was randomly determined. We observed that all the controls gave rise to similar results; hence, all the analysis in the main text is based on the fifth control data set.

### Characteristic quantities in the context of module

For each gene involved in at least one module, we defined six corresponding characteristic quantities in the context of module. Without loss of generality, we assumed that a gene i (g_i_) participates in n modules 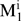,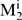,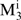,… and 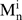, where the superscript refers to the corresponding gene and the symbol M_i_ denotes the module M_i_ (G_i_, T_i_) as defined before. Note that T_i_ includes only those tissues which have a positive tissue score and a module 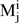 is counted only if its corresponding T_j_ is not null. The six variables are formulized as:

1. Number of modules = n, which define the number of modules which contain the corresponding gene.
2. Number of interactors = |
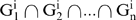
|, which count how many neighbor genes interact with the corresponding gene.
3. Number of interactions = |
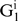
|+|
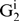
|+…+|
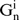
|, which specify how many interactions between the neighbor genes and the corresponding gene. This can be considered as the “weighted” version of “Number of interactors”.
4. Number of tissues = |
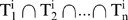
|, which measure the number of tissues in which the corresponding gene is highly expressed.
5. Number of tissues (repeated) = |
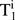
|+|
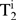
|+…+|
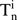
|, which may be viewed as the “repeatable” Number of tissues. Note: a tissue is counted k times only if it is associated with k different modules which contain the corresponding gene.
6. Total constraint intensity = |
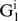
|
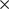
|
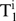
|+|
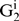
|
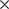
|
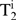
|+…+|
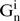
|
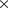
|
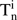
|, which calculates the total constraint force on a gene as the summation of the constraint intensity exerted by each module. And the constraint force of a module upon a gene is conducted as the product of the size of corresponding gene set and that of the corresponding tissue set.

### Calculation of Ka/Ks

All the sequences of protein-coding genes of human (Build NCBI36), mouse (Build NCBI37) and cow (Build NCBI3.1) were retrieved from the Ensembl website [45]. The human-mouse-cow orthologous (HMC triplex) relationship is specified by a mapping file downloaded with the use of the BioMart tool [46]. For each HMC triplex, we run the transAlign.pl script [47] which implicitly invokes the ClustalW [48] tool to output aligned sequences. Then, for each aligned HMC triplex, we infer the human-mouse ancestral sequence using the cow ortholog as outgroup by the baseml program [49] implemented in the PAML package [50]. Synonymous (Ks) and nonsynonymous (Ka) substitution rates were calculated for alignments of protein-coding sequences using the LPB93 method [51] imbedded in the yn00 program [52]. The lineage-specific Ka/Ks ratios were computed by the comparison between the inferred sequences at the human–mouse ancestral node and the sequences at the human or mouse node.

### Gene ontology analysis

GO provides three controlled vocabularies (ontologies) that describe gene products in terms of their associated biological processes, cellular components and molecular functions into structured directed acyclic graphs (DAGs) [53]. To determine the enriched GO terms of genes within a module, we conducted GO enrichment analysis using the GO Term Finder package [37]. GO annotation files were downloaded from ftp://ftp.ebi.ac.uk/pub/databases/GO/goa/ on December 10, 2008. GO ontology file was downloaded from http://www.geneontology.org/ on December 22, 2008.

### Sensitivity test of modules

We created four groups of datasets (each group includes 20 human and 20 mouse gene expression matrixes) by randomly removing 5%, 10%, 15% and 20% genes of the original datasets, we then identified the modules using the above-mentioned approach. For each dataset, we obtained a similarity matrix by calculating the pair-wise similarities by Eqa. **(1)**. between the whole modules and those identified when the full data were used given Tc = 1.5 and Tg = 3.0. For the similarity matrix, we got the maximal similarity value row-by-row (if the number of rows is less than that of columns, otherwise we transpose the matrix) and computed their mean (*S*). Then for each group, we calculated the mean and the standard deviation of *S*, from which the robustness of modules was evaluated.

## Supporting Information

Table S1 List of 52 human modules.
(0.04 MB DOC)

Table S2 List of 65 mouse modules.
(0.05 MB DOC)

Table S3 Overlapped GO functional terms in five pairs of inter-species modules.
(0.04 MB DOC)

Figure S1 Gene expression pattern across tissues. The y-axis value is the logarithm of the gene expression level to the base 10.
(1.54 MB TIF)

Figure S2 Relationship between the number of modules and the ISA thresholds used. (A) Human; (B) mouse. The number of modules is proportional to the area of the “Ball.”
(0.14 MB TIF)

Figure S3 Similarity of modules within and between species. The heat map prominently displays a highly low similarity of modules from between species in contrast to those within each species. Rows and columns numbered 0–51 and 52–116 represent the human and mouse modules, respectively.
(0.42 MB TIF)

Figure S4 Hierarchical clustering graph of 117 (52 human and 65 mouse) modules. The tree indicates that only few pairs of the modules, which are derived from the two species respectively, have a relatively high overlap of genes. The filled cycles denote the human modules, and the unfilled cycles denote the human mouse modules.
(0.87 MB TIF)

Figure S5 The statistical significance of the observed maximal similarity. The plot shows that a majarity of the interspecies modules have a low gene overlap. Note that a largest maximial simialrity (0.451) is not shown only for aesthetics.
(0.47 MB TIF)

Figure S6 Histograph of the Ks in human or mouse lineage.
(0.11 MB TIF)
