# Supplementary material for: Characterization and Comparison of the Tissue-Related Modules in Human and Mouse
Source: PLoS One. 2010 Jul 22;5(7):e11730. doi: 10.1371/journal.pone.0011730 (PMC2908688; doi:10.1371/journal.pone.0011730)
Supplement: Table S1 — List of 52 human modules. (0.04 MB DOC) [file pone.0011730.s001.doc]

1. HSD17B2 WFDC1 SERPINE1 MGP SPARC IGFBP5 GNG11 GDF15 TBX3 THBS1 TIMP2 NID1 LUM ADM ESAM MYL9 HSPB8 CG38_HUMAN C1orf115 CLEC3B CD14 SDC2 PMP22 C8orf4 FBLN1 PAPPA KRT7 F3 CAV1 PPL PTRF COL4A2 WWTR1 KRT19 TCF21 CTGF C6orf189 TIE1 MAFB CLIC4 :::::::: CD4+Tcells CD8+Tcells placenta
2. UQCRC1 COPE CLDN3 DCI RFXANK DCXR PPP1CA PRPF31 SG196_HUMAN KRT7 PPL MAFB PRKCZ :::::::: dorsal root ganglion
3. UQCRC1 CORO1A NKG7 AGR2 LTF KRT15 COPE ELA2 ATBF1 SLC20A1 NCAPD3 CA2 HOXB13 RNF149 CAMP CLDN3 CANT1 RFXANK BCL2L1 PRG2 PPP1CA PRPF31 SG196_HUMAN KRT7 SLC16A3 MAP7 IGF2R MRPS16 MAFB CD52 PAK1IP1 :::::::: bone marrow dorsal root ganglion ovary prostate trigeminal ganglion
4. UQCRC1 NME3 CKM AGR2 LTF TNNC1 KRT5 GAMT CNN1 GDF15 TRPM4 KRT15 TBX3 COPE SORD ABAT ATBF1 NCAPD3 HOXB13 PIP PRKD2 TNNT1 AZGP1 TGM4 CLDN3 MESP1 ANPEP DCI CANT1 DCXR PPP1CA PRPF31 CD151 KRT7 HEXIM1 SPINT1 MAP7 KRT19 ACTA1 TACSTD2 GNMT MAFB DES SPDEF PRKCZ PAK1IP1 NKX3-1 :::::::: dorsal root ganglion ovary prostate
5. UQCRC1 PTGDS RND2 TNP1 CCDC53 DYNLL1 MEA1 HSPA2 SYNGR3 COPE NPTX2 ABAT UBIQ_HUMAN SF3B2 NDUFA12 TMEM130 MAP7 CGA PRKCZ POMC :::::::: dorsal root ganglion hypothalamus ovary pituitary testis
6. UQCRC1 DYNLL1 HSPA2 SYNGR3 GDF15 DYSF SERPINE2 SLC35A5 ABAT SLC20A1 HSPB8 CG38_HUMAN C1orf115 MOAP1 PRG2 KRT7 HEXIM1 TUBB2A SLC38A1 CALM1 CGA PRKCZ C13orf15 AVP :::::::: hypothalamus ovary placenta
7. YPEL3 CORO1A NKG7 LTA4H ARHGDIB LTF SRGN LENG4 RAC2 LMO2 CYBA BLVRB ELA2 VAMP8 IMPA2 NRGN CA2 LAPTM5 RNF149 PPBP TKT CAMP CD14 NINJ2 BCL2L1 PRG2 PPP1CA FLII SG196_HUMAN P4HB RNH1 IGF2R G0S2 CITED2 S100A8 S100A9 CD52 CST7 :::::::: bone marrow dorsal root ganglion lung trigeminal ganglion
8. APOH SERPIND1 RARRES2 VTN HRG REEP6 TTR C3 GLTSCR2 APOE APOC2 APOC1 COX4I1 APCS ALDH2 RPL8 SLC27A5 TF AMBP HPX ITIH1 AHSG GC SERPING1 NNMT CD14 UBIQ_HUMAN DCXR AGXT EEF2 F2 CLU RPLP2 P4HB ACTB UBIQ_HUMAN CYP1A2 FXYD1 AGT APOA2 ECHS1 MAT1A FBP1 :::::::: liver
9. HOXA11 PDCD7 NP_683701.2 ATXN1 CNN1 MRPL34 CCDC124 AKAP8L HAT1 NID1 SLC22A3 CCT8 C7orf30 AGPAT6 ZHX1 PDIA3 ACOX2 FAM18B SH3YL1 ZFPM2 WT1 NP_068761.4 C5orf24 SMOC2 PELI1 CITED2 EMX2 STAG2 COL5A2 LRRC47 EMILIN1 :::::::: trigeminal ganglion uterus
10. LGALS1 ACP5 FCGRT NKG7 RARRES2 CSDA MGP TNNC1 RRAS CNN1 VAMP8 IMPA2 TAGLN MYL9 CLEC3B MFAP4 CD151 CFD FBLN1 SG196_HUMAN P4HB CAV1 TCF21 S100A9 DES CD52 :::::::: amygdala cerebellum lung
11. C16orf42 KCNQ1 NME3 TG C2orf40 GSTT1 IGJ SLC35A5 SGK3 COPE HIRA CPVL CPEB3 ABAT PTH KCNJ16 TFF3 ZER1 ADCY9 CLDN3 MOAP1 CRABP1 COQ4 DCI KCNAB1 BCL2L1 STK25 PRPF31 SG223_HUMAN SG196_HUMAN KRT7 SLC16A3 ALDH5A1 FOSB MAP7 CDC42BPB CDC73 NINJ1 PRKCZ C13orf15 :::::::: ovary thyroid tongue
12. EEF1A2 STMN2 APLP1 PTGDS SPARC TTR HSPA2 SYNGR3 APOE TUBB2B SYT1 TF FEZ1 GPM6A SPOCK1 SPARCL1 ZIC1 DDAH1 CHN1 GRIA2 NDRG2 SCG5 GPRC5B NSG2_HUMAN PMP22 CLU DKK3 PCP4 SV2B TUBB2A DNER APOD CKB CLIP3 STXBP1 CAMK2N1 CST3 :::::::: CD4+Tcells CD8+Tcells amygdala cerebellum hypothalamus
13. CRLF1 UQCRC1 COMP LTA4H LTBR TBX3 THBS1 TIMP2 COPE NID1 POR IMPA2 SLC20A1 ADFP PDIA3 DCXR PPP1CA CD151 FLII CFD SG196_HUMAN P4HB KRT7 PPL AKT1 RNH1 IGF2R UNC50 COL4A2 KRT19 COL6A1 CITED2 XPNPEP1 MAFB MFAP2 LRRC47 PRKCZ :::::::: adipocyte dorsal root ganglion lung placenta trigeminal ganglion
14. CORO1A ARHGDIB GLTSCR2 RAC2 BTG1 RPL8 RPL32 RPS3 RPL30 LAPTM5 CD3D IL7R RPS7 RPLP2 ACTB NBEAL1 CD52 :::::::: CD4+Tcells CD8+Tcells
15. CRY1 ASNS PGRMC1 LRP11 CCDC53 BHLHB3 ATXN1 DHX29 PPIL4 WDR7 SLC35A5 SGK3 LY75 TOP2B IQGAP1 ABAT CDR2 SLC39A6 PTH TIA1 CCT8 CDS1 ZHX1 CLDN3 SEC11C PDIA3 KIAA1826 SDC2 EIF2AK3 SCG2 FAM18B SH3YL1 DMXL1 RECQL SPAST PRPF3 KRT7 NP_068761.4 C5orf24 WDR22 PPL ALDH5A1 COG3 MAP7 UNC50 HIBCH PJA2 AKT3 EDEM3 CGA AHCYL1 CETN2 STAG2 CD2AP NAP1L3 YIPF6 LGR4 POMC :::::::: heart pituitary thyroid trigeminal ganglion
16. CRY1 PGRMC1 BHLHB3 TBX3 TOP2B SORD SLC39A6 CCT8 CDS1 ZHX1 CLDN3 SEC11C PDIA3 FAM18B SH3YL1 DMXL1 SG196_HUMAN KRT7 CLDN4 ALDH5A1 MAP7 SLC2A10 KRT19 CDC73 STAG2 TACSTD2 TSPAN1 LGR4 :::::::: heart prostate thyroid trigeminal ganglion
17. CRY1 LRP11 SRGN BHLHB3 PPIL4 PNPLA10P GLCE SGK3 TOP2B IFT57 LUM IQGAP1 SLC39A6 GPM6A PTH TIA1 CDS1 BRD7 KCNAB1 SCG2 FAM18B DMXL1 RECQL SPAST CLK1 PRPF3 ZNF292 MAP7 KTN1 MYT1 AKT3 CGA CETN2 STAG2 CD2AP NAP1L3 DDX50 POMC MTMR6 :::::::: heart liver pituitary thyroid
18. NME3 CADM4 AGR2 SART3 TNNC1 PPP1R1A SGK3 COPE VPS37B ABAT MRPS28 WIF1 PDE6D CLDN3 SAC3D1 TAPT1 COG7 SPAST PRPF31 ATP13A2 MAP7 ENDOG PRKCZ PAK1IP1 :::::::: dorsal root ganglion hypothalamus ovary prostate salivary gland
19. PGLYRP1 UQCRC1 CORO1A CKM NKG7 ARHGDIB LTF TNNC1 CFP GAMT DYSF SLC4A1 COPE BLVRB ELA2 LYL1 CSRP3 MS4A3 NRGN CA2 CCDC12 PPBP CAMP APEH RFXANK BCL2L1 EHBP1L1 ALAS1 PRG2 PPP1CA SLC35A4 FLII SG196_HUMAN DGAT1 GFI1B SLC16A3 IGF2R MAPKAPK3 MPDU1 G0S2 S100A9 MRPS16 MRPS15 MAFB CD52 CDA NINJ1 CST7 :::::::: bone marrow dorsal root ganglion heart ovary trigeminal ganglion
20. WFDC1 AGR2 MGP SPARC LTF IGFBP5 NP_683701.2 CNN1 GDF15 TRPM4 KRT15 TBX3 TANC1 PDZRN3 LUM ATBF1 ZNF750 SLC22A3 MYL9 HOXB13 AZGP1 TGM4 CLDN3 MESP1 SDC2 C8orf4 CD151 KRT7 CAV1 PPL MAP7 WWTR1 KRT19 TACSTD2 MAFB CLIC4 :::::::: CD4+Tcells CD8+Tcells prostate
21. MGST1 WFDC1 RARRES2 MGP SPARC AADAC IGFBP5 RAB38 C3 LDLR APOE APOC1 TBX3 DBH NID1 NPTX2 LUM NP_060712.2 STAR MYL9 SLC16A9 CD14 C1S SERPINA5 FADS1 CYP17A1 CLIC4 CP21A_HUMAN :::::::: CD4+Tcells CD8+Tcells adrenal gland
22. STMN2 APLP1 PTGDS ENO2 TTR DYNLL1 SYNGR3 APOE SYT1 TF FEZ1 GPRC5B UBIQ_HUMAN CLU ACTB ACTG1 TUBB2A KIF5C CALM1 STXBP1 CAMK2N1 :::::::: amygdala cerebellum hypothalamus skeletal muscle
23. CKM MGP SPARC TNNC1 IGFBP5 C3 APOE APOC1 CSRP3 MYL9 HSPB8 MYL3 COX7A1 CLEC3B MFAP4 CD14 HSPB2 PMP22 C1QB CD151 CFD CLDN5 CAV1 PTRF TCF21 TIE1 MAFB :::::::: CD4+Tcells CD8+Tcells heart lung
24. CKM MGP SPARC IGFBP5 BHLHB3 PPP1R1A TANC1 CSRP3 LUM ZNF750 MYL9 CLDN3 SDC2 C8orf4 KRT7 CAV1 MAP7 WWTR1 CDC42BPB MAFB CLIC4 ID4 :::::::: CD4+Tcells CD8+Tcells thyroid
25. APLP1 CCT8 SCG5 UBIQ_HUMAN SCG2 CIRBP EIF4A2 UBIQ_HUMAN CGA CHGB POMC :::::::: hypothalamus pituitary trigeminal ganglion
26. APLP1 LTA4H EBNA1BP2 ATXN1 MRPL34 FSHB WDR7 AKAP8L NPTX2 CCT8 C7orf30 APEH GRIA2 CLDN3 PDIA3 C1QTNF4 SCG2 FAM18B SH3YL1 FKBP2 CIRBP NP_068761.4 PPL UNC50 PELI1 CGA CEL YIPF6 LRRC47 CHGB POMC :::::::: pituitary trigeminal ganglion
27. GPRC5A KCNQ1 UQCRC1 ACP5 RUVBL2 NKG7 DUSP1 APOC1 C20orf114 Q5FWF1_HUMAN COPE LAMP3 SCGB1A1 AYTL2 NRGN LY6E ADCY9 RNF149 CLEC3B CLDN3 DCI SAC3D1 RFXANK NINJ2 DCXR PPP1CA SFTPC PRPF31 CD151 MARCO GIMAP6 CLDN5 SG196_HUMAN KRT7 HEY1 PPL KRT19 TACSTD2 SFTPD TIE1 MAFB CD52 FBP1 PRKCZ C13orf15 EDN1 :::::::: dorsal root ganglion lung ovary
28. GPRC5A KCNQ1 GADD45B NME3 RUVBL2 FCGRT TNNC1 DUSP1 PRKCSH JUND CYBA COPE SIL1 POR TFF3 CLEC3B CLDN3 DCI DCXR SFTPC PRPF31 CFD NP_001111.2 CLDN5 THOC4 KRT7 RNH1 CITED2 MAFB NINJ1 PRKCZ :::::::: dorsal root ganglion lung salivary gland thyroid
29. GPRC5A KCNQ1 GADD45B FCGRT DUSP1 CYBA COPE HIRA ADFP SCGB1A1 MYL9 ADCY9 CLDN3 FOS SFTPC PRPF31 GIMAP6 CLDN5 SG196_HUMAN KRT7 AKT1 IGF2R CITED2 PRKCZ C13orf15 :::::::: dorsal root ganglion lung thyroid tongue
30. VIM GDF15 CD63 SERPINE2 CYP19A1 TIMP2 IGFBP1 UBIQ_HUMAN PRG2 PAPPA ACTB ACTG1 UBIQ_HUMAN CGA :::::::: placenta
31. MGP SPARC IGFBP5 CNN1 TANC1 PDZRN3 NID1 LUM SLC22A3 MYL9 SDC2 C8orf4 CAV1 STK3 WWTR1 KRT19 EMX2 CLIC4 :::::::: CD4+Tcells CD8+Tcells uterus
32. MGP SPARC IGFBP5 ATP6V1B1 APOE GDF15 PPP1R1A ENPEP MYL9 AZGP1 PDZK1IP1 FABP1 SLC16A9 CDH16 SDC2 UMOD GATM C8orf4 PCK1 HSD11B2 SERPINA5 CXCL14 DPYS MAP7 WWTR1 NPHS2 EMX2 XPNPEP2 CLIC4 ALDOB KL :::::::: CD4+Tcells CD8+Tcells kidney
33. MGP SPARC IGFBP5 C3 LUM MYL9 CLEC3B MFAP4 PMP22 C8orf4 CD151 CLDN5 KRT7 F3 CAV1 PPL WWTR1 KRT19 MAFB CLIC4 :::::::: CD4+Tcells CD8+Tcells lung
34. LTF CLTA GLTSCR2 CNN1 C20orf114 SCGB1A1 MYL9 TFF3 CSTB PPIB PDIA3 FOS SG196_HUMAN P4HB PLXNB2 KRT19 TACSTD2 :::::::: lung skeletal muscle trachea
35. LCP2 KRT14 ARHGDIB CDCA3 LTF CXCR4 ZFP36 RAC2 KRT5 C20orf114 KRT15 FABP4 CTSL2 SIT1 LY75 VAMP8 ZAP70 CPVL FBLN5 MYL9 PRKD2 CD3G RAG1 CD3D SAC3D1 CD7 EZH2 CFD THOC4 SG196_HUMAN RRM2 KRT19 S100A9 CD2 DNTT ZNF217 CEP55 ADA CD52 AIF1 :::::::: amygdala cerebellum thymus
36. GRN PRKCSH JUND TIMP2 COPE POR ESAM CLEC3B KRT7 CAV1 RNH1 COL4A2 PRKCZ :::::::: dorsal root ganglion lung placenta salivary gland thyroid
37. GRN GDF15 CLEC3B UBIQ_HUMAN SFTPC OAZ1 SG196_HUMAN KRT7 LAMP1 KRT19 PRKCZ C13orf15 :::::::: lung ovary placenta
38. GRN HSD17B2 UQCRC1 GDF15 TIMP2 COPE VAMP8 SLC20A1 ADFP ADM ESAM HSPB8 C1orf115 CLEC3B CD14 PRG2 PAPPA SG196_HUMAN P4HB KRT7 HEXIM1 CDH5 KRT19 CGA TIE1 MAFB PRKCZ C13orf15 :::::::: dorsal root ganglion lung ovary placenta
39. GRN GADD45B NKG7 RARRES2 AGR2 CSDA LTBR LTF TNNC1 DUSP1 GLTSCR2 CNN1 GDF15 TBX3 COPE VAMP8 SIL1 IMPA2 ADFP SCGB1A1 MYL9 PRKD2 CLEC3B CLDN3 MFAP4 DCXR SFTPC CD151 CFD GIMAP6 SG196_HUMAN P4HB KRT7 CAV1 CYR61 KRT19 TCF21 TACSTD2 MAFB DES CD52 FBP1 :::::::: cerebellum dorsal root ganglion lung prostate
40. GRN ACP5 LTA4H DUSP1 GLTSCR2 APOC1 MRPL34 C20orf114 SCGB1A1 LY6E CLEC3B DCXR SFTPC CFD CLDN5 SG196_HUMAN P4HB KRT7 CDH5 RNH1 PLXNB2 TACSTD2 SFTPD CD52 FBP1 PRKCZ EDN1 :::::::: lung trigeminal ganglion
41. GRN NME3 AGR2 TNNC1 DUSP1 PRKCSH CNN1 JUND COPE SIL1 POR SCGB1A1 MYL9 CLEC3B CLDN3 PPP1R14A DCXR SFTPC PRPF31 CFD NP_001111.2 CLDN5 THOC4 AKT1 RNH1 KRT19 TCF21 PGC DES PRKCZ :::::::: dorsal root ganglion lung prostate salivary gland
42. GRN RARRES2 GLTSCR2 APOE CYBA EMP3 MYL9 CSTB CLEC3B MFAP4 PPIB C1QB SFTPC CRIP2 SG196_HUMAN P4HB PLXNB2 CTGF C13orf15 :::::::: lung skeletal muscle
43. KCNQ1 UQCRC1 NME3 TG CKM TNNC1 AHR MYH2 PPP1R1A SLC35A5 SGK3 COPE HIRA LY75 POR CSRP3 CPEB3 ABAT PTH ZER1 TNNT1 ADCY9 CLDN3 COQ4 DCI SH3YL1 TCAP PPP1CA STK25 NP_037424.2 PIB5PA SG196_HUMAN KRT7 PPL ALDH5A1 MAP7 MYBPC2 CDC42BPB ACTA1 MAFB PRKCZ C13orf15 :::::::: ovary thyroid trigeminal ganglion
44. KCNQ1 UQCRC1 NME3 RUVBL2 CKM NKG7 TNNC1 MRPL34 LRP16_HUMAN PPP1R1A COPE CSRP3 ADCY9 CLEC3B APEH CLDN3 DCI RFXANK DCXR SSSCA1 PPP1CA PRPF31 CD151 COX5A SLC35A4 CFD KRT7 DGAT1 AKT1 ACTA1 TIE1 ENDOG MRPS15 MAFB DES LRRC47 PRKCZ :::::::: dorsal root ganglion heart ovary trigeminal ganglion
45. KCNQ1 UQCRC1 NME3 DUSP1 MRPL34 COPE SCGB1A1 ADCY9 CLEC3B CLDN3 DCI DCXR PPP1CA STK25 SFTPC CD151 CFD CLDN5 THOC4 SG196_HUMAN KRT7 CDH5 PPL PSMB10 CDC42BPB EFNA1 SFTPD TIE1 MAFB PRKCZ C13orf15 :::::::: lung ovary trigeminal ganglion
46. KCNQ1 UQCRC1 RARRES2 PPIF LTA4H AADAC LENG4 LDLR APOC1 BST2 TBX3 COPE DBH SIL1 POR SLC22A3 STAR CCDC12 FDXR APEH CD14 IRF2 FAM18B FKBP2 ALAS1 DAK MRPS23 ST8SIA5 FLII KLHL22 DGAT1 KLF12 CCDC69 IGF2R UNC50 CITED2 CYP17A1 TIE1 CP21A_HUMAN :::::::: adrenal gland dorsal root ganglion trigeminal ganglion
47. KCNQ1 UQCRC1 DUSP1 COPE SIL1 POR CLDN3 DCI DCXR FKBP2 PPP1CA FLII SG196_HUMAN KRT7 PPL AKT1 IGF2R CITED2 MAFB PRKCZ :::::::: dorsal root ganglion lung thyroid trigeminal ganglion
48. KCNQ1 GADD45B TG CKM CSDA MYL2 MGP TNNC1 MYOT MYH2 ZFP36 IGJ MATN2 TBX3 VAMP8 SIL1 POR CPVL SLC26A4 CSRP3 LUM SORD IMPA2 MYL9 PTH HGD PRKD2 TFF3 TNNT1 CLDN3 CDH16 COQ4 DCI TCAP NP_037424.2 CFD FBLN1 PIB5PA SG196_HUMAN KRT7 CAV1 MYBPC2 MB CYR61 ACTA1 MAFB DES SMPX :::::::: amygdala cerebellum thyroid
49. KCNQ1 PGRMC1 NME3 NOLA1 ENO2 C2orf40 APOE PEMT SGK3 ABAT MYL9 TFF3 CLDN3 PPIB PDIA3 SDC2 KCNAB1 FOS FAM18B FKBP2 B3GALNT1 SG196_HUMAN MAP7 SPG20 PLD3 UNC50 AKT3 STAG2 NAP1L3 C13orf15 POMC :::::::: pituitary skeletal muscle thyroid tongue
50. KCNQ1 NME3 C2orf40 COPE TFF3 CSTB ADCY9 CLEC3B CLDN3 PPIB FOS PPP1CA SFTPC CRIP2 SG196_HUMAN KRT7 PLXNB2 CDC42BPB CTGF EFNA1 MAFB PRKCZ C13orf15 :::::::: lung ovary skeletal muscle thyroid
51. KCNQ1 TG CKM MYH2 MRPL34 POR PTH CLDN3 PDIA3 DCI SDC2 SH3YL1 CIRBP NP_037424.2 CFD SG196_HUMAN KRT7 SMOC2 ACTA1 CITED2 LRRC47 :::::::: thyroid trigeminal ganglion
52. KCNQ1 TG C2orf40 HIRA MYL9 PTH KCNJ16 TFF3 CLDN3 PDIA3 SDC2 FOS KRT7 CITED2 :::::::: thyroid tongue
