# Supplementary material for: Characterization and Comparison of the Tissue-Related Modules in Human and Mouse
Source: PLoS One. 2010 Jul 22;5(7):e11730. doi: 10.1371/journal.pone.0011730 (PMC2908688; doi:10.1371/journal.pone.0011730)
Supplement: Table S2 — List of 65 mouse modules. (0.05 MB DOC) [file pone.0011730.s002.doc]

1. Hspb6 Pdk4 Dcn Pvalb Eef1a2 Mt4 Ckm Mgp Sparc Igfbp5 Myot Apobec2 C3 Krt13 Hrc Krt15 Myh4 Fabp4 Cd63 Nid1 1110028A07Rik Rpl3l Hspb8 Sparcl1 Cox6a2 Cox7a1 Krt4 Tmod4 Cmbl Col1a2 Pgam2 Ndrg2 Sdpr Rnase4 Ckmt2 1810011O10Rik Mylpf Cfd Cyp2f2 Bgn Krtdap Pfkm Cav1 Plekhc1 Ppl Thbs4 Fxyd1 Atp2a1 Myoz1 Mb Acta1 Dpt Casq1 Crct1 Gsta4 zfp503 Des Aspn Smpx Dsp :::::::: CD4+Tcells CD8+Tcells skeletal muscle tongue trachea
2. Pdk4 Acp5 Bcl6 Acadl Igfbp5 Ppp1r3c Myot C3 Apoe Myh4 Fabp4 Serpine2 Bzw2 Trf Tmem45b Hspb8 Ndrg2 Tmem43 Pck1 Mylpf Hrasls3 Cfd Cyp2f2 Ktn1 Mb Acta1 Dpt Psmb9 :::::::: adipocyte pancreas skeletal muscle testis trachea
3. P2ry10 Coro1a Apoe Lgals3 Btg1 Cyba Ncor1 Dck Rag1 9030416H16Rik Ets1 2-Mar Cep57 Tubb2a Lrmp Smc4 Rrm2 S100a9 Hprt1 Dntt Psmb9 :::::::: bone marrow pancreas thymus
4. P2ry10 Srd5a2 Tmem45b Tmem100 Snx7 Cep57 Pak2 Arl6 AB112350 Ktn1 Zmym1 Hemgn Serpinb5 :::::::: adrenal gland liver pancreas
5. Ptpn18 Ccdc80 Acp5 Ehhadh Acadl Cat C3 Apoe Lgals3 Fabp4 Cyba Trf Prkar2b Tmem45b Ly6e Ccl5 Tmem43 Tlr1 Ptpn6 Pck1 Adipoq Hrasls3 Cfd Cyp2f2 F3 Slc36a2 Ms4a1 Tmem123 Krt19 Acta1 G0s2 Dpt Psmb9 :::::::: adipocyte pancreas testis trachea
6. Ptpn18 Rg9mtd2 Krt23 Acp5 Mt4 Pon1 Agr2 Gimap1 Icam2 Mgp Lama4 Ehhadh C3 Krt13 Zfp36 Apoc1 U46068 Krt15 Lgals3 Fabp4 Xlkd1 Emp1 Chad Cyba Crispld2 Birc3 Ifih1 Calcrl Zfp750 Scgb1a1 Ly6e Ccl5 Aqp5 Krt4 Pcolce2 Aqp3 Mfap4 Sdpr Rnase4 Lrg1 1810011O10Rik Trim25 Ptpn6 Pck1 Marco Cfd Gimap6 Cyp2f2 Krtdap Gvin1 Cav1 Colec12 Ms4a1 Ppl Pigr Lypd2 Mb Krt19 G0s2 Dpt S100a14 Crct1 Mical1 Gpc3 Tacstd2 Ada Psmb9 Itga8 Xdh Dsp Emilin1 :::::::: amygdala cerebellum lung testis trachea
7. Ptpn18 Acp5 Gimap1 Cd69 Ehhadh Prtn3 C3 Indo Fabp4 Il5ra Cyba Ifih1 Wnt2 Cd226 Tmem45b C1r Osr2 Slfn5 Slamf1 Lrg1 Tlr1 Gpr171 1810011O10Rik Ptpn6 Pck1 BC038479 Gvin1 Nr1h3 Ms4a1 Ptrf Krt19 G0s2 Mical1 Tacstd2 Tie1 Psmb9 Was :::::::: adipocyte amygdala cerebellum hypothalamus olfactory bulb testis
8. Ptpn18 Coro1a Lgals3 Myh4 Cyba Glipr1 Dck Slc25a37 Ly6e Rag1 Cdt1 Tmem43 Ptpn6 Ets1 Trak2 Tubb2a Lrmp Rrm2 Acta1 S100a8 S100a9 Hprt1 Dntt Psmb9 Hemgn Was :::::::: bone marrow pancreas testis thymus
9. Esr1 Apoe Lgals3 Mfap4 Tmem43 2-Mar Cep57 Pcp4 Grb10 Dpysl3 Krt19 :::::::: ovary pancreas uterus
10. Esr1 Acp5 Lama4 1700025G04Rik Cfp Lgals3 Matn2 Serpine2 Cyba Calcrl Nid1 Trf Nedd1 Tmem45b Dkk2 Ndrg2 Tmem100 Ppp1r14a Ndufa7 Nudt9 Hoxc9 Tmem43 Tlr1 Cyfip1 B3galnt1 Cep57 Pcp4 Tubb2a F3 Slc36a2 Grb10 Arl6 2010310D06Rik BC038479 Bzw1 Cmtm3 Tmem23 Krt19 Pex3 Aox1 Psmb9 :::::::: adipocyte ovary pancreas salivary gland
11. Esr1 Chd9 Pdcd5 Ehhadh 1700025G04Rik Fshb Ccdc59 Tshb Ncor1 Cdh2 D230037D09Rik Slc6a1 Ppp1r14a Tmem43 B3galnt1 Cep57 Pcp4 Ndn Tubb2a Arl6 Rnf13 Spock3 Tmem23 Stxbp1 Bmi1 Serpinb5 :::::::: pancreas pituitary
12. Esr1 Dctn6 Parp2 Tshb Tmem100 Ppp1r14a Tmem43 B3galnt1 Cep57 Pcp4 Arl6 Ktn1 Taf11 Tmem23 Ilf2 :::::::: liver pancreas pituitary
13. Esr1 Agr2 Ehhadh Prrg2 Fshb Sec14l4 Il5ra Tshb Ifih1 Pitx1 Zfp750 Osr2 Entpd3 Cdkn2aip Klk7 2310032F03Rik 1810011O10Rik Tmem30b Tcte3 Psmb9 Galnt12 Grpr Esm1 :::::::: amygdala cerebellum hypothalamus pituitary
14. Esr1 Gimap1 Ehhadh Zc3h13 Matn2 Calcrl Tmem45b Tmem100 Tmem43 Snx7 Tlr1 Cep57 Pak2 F3 Psmb9 Hemgn :::::::: pancreas testis
15. Esr1 Gimap1 Zc3h13 Serpine2 Calcrl Nedd1 Tmem45b Tmem100 Ppp1r14a Hoxc9 Snx7 Tlr1 Cyfip1 B3galnt1 Ruvbl1 Cep57 Tubb2a F3 Arl6 2010310D06Rik BC038479 AB112350 Thbs4 Zmym1 Ilf2 Crabp2 Cyp17a1 Lect1 Emilin1 :::::::: ovary pancreas salivary gland thyroid
16. Esr1 Aebp1 Lama4 Msh2 1700025G04Rik C3 Matn2 Serpine2 Bzw2 Cyba Upf1 Calcrl Nedd1 2610027C15Rik Adamts19 Tmem45b Osr2 Gas1 Tmem100 Mfap4 Ppp1r14a Ndufa7 Tmem43 Snx7 Spon1 Cyfip1 Tubb6 B3galnt1 Ruvbl1 Cep57 Pcp4 Marcksl1 Tubb2a F3 Grb10 Arl6 2010310D06Rik Smoc2 Cmtm3 Krt19 Crabp2 Thbs3 Tacstd2 Psmb9 Emilin1 :::::::: ovary pancreas salivary gland thyroid uterus
17. Esr1 Lama4 1700025G04Rik Zc3h13 Snx9 Matn2 Serpine2 Rock1 Cyba Calcrl Nid1 Trf Tmem45b Cstb Tmem100 Ppp1r14a Nudt9 Tmem43 Snx7 Sema3e Hrb Spon1 Cyfip1 B3galnt1 Cep57 Marcksl1 Tubb2a F3 Grb10 Arl6 2010310D06Rik Bzw1 Bicd2 Zmym1 Cmtm3 Krt19 Cyp17a1 Gpc3 Guca2b Lect1 Emilin1 :::::::: ovary pancreas placenta salivary gland thyroid
18. Esr1 Ehhadh Lgals3 Ccdc59 Calcrl Nedd1 Ncor1 Tmem45b Tmem100 Ppp1r14a Tmem43 Snx7 Cep57 Pak2 Arl6 Rnf13 Tmem23 Cyp17a1 Bmi1 Lect1 :::::::: ovary pancreas
19. Esr1 Ehhadh Zc3h13 Fshb Hrsp12 Matn2 Tshb Serpine2 D230037D09Rik Slc6a1 Tmem43 Slitrk6 Cep57 Pak2 Pcp4 Tubb2a Otx2 Ints8 Ktn1 Spock3 Ilf2 Synpo2l Psmb9 Serpinb5 :::::::: pancreas pituitary testis
20. Rg9mtd2 Srd5a2 C3 BC054438 Matn2 Fabp4 Camk1 C530044N13Rik Rasgrf1 Pdgfrb Nid1 Pnmt Tmem45b Hspb8 Flt1 Steap2 C1r Nr2f6 Cckar Cmbl Osr2 Sdpr Scg2 Snx7 Lrrn3 1810011O10Rik Pck1 Bcan Ndn F3 AB112350 Colec12 2900041A09Rik G0s2 Gpc3 Bmp7 Tie1 zfp503 Chgb Esm1 :::::::: CD4+Tcells CD8+Tcells adrenal gland testis
21. Tcn2 Afp Ehhadh Prg1 Lgals3 Acy3 Cd63 Serpine2 Hexb Cyba Trf Tspan14 Slc27a2 Cstb Car4 Tmem43 Pck1 Hprt1 Slc2a1 Guca2b Cda Apom Gjb2 :::::::: kidney pancreas placenta testis
22. Cd79b Dcn Ptpn18 Lgals1 Acp5 Vim C3 Cfp Lgals3 Fabp4 Xlkd1 Cyba Cnn2 Stk17b Nid1 Cxcl13 Ccl5 Col1a2 Ly6d Lrg1 Slc1a5 Tlr1 Ptpn6 Pck1 Adipoq Marco Cfd EG317677 Ms4a1 Tmem123 Faim3 Dpt Cd52 Psmb9 :::::::: adipocyte amygdala hypothalamus trachea
23. Pvalb Coro1a Ckm Jund1 Lgals3 Myh4 Slc4a1 Car2 Slc25a37 Ppbp Camp Ubc Rplp2 Oaz1 Mylpf Atp2a1 Acta1 S100a8 S100a9 Hemgn Rsad2 :::::::: bone marrow pancreas
24. Pigh Afp 1700025G04Rik Fshb Lgals3 Tshb Hexb Glipr1 D230037D09Rik Tmem100 Ppp1r14a Tmem43 Sema3e Gga2 B3galnt1 Cep57 Grb10 Arl6 Ktn1 Zmym1 Tmem23 Serpinb5 :::::::: liver pancreas pituitary placenta
25. Eef1a2 Scg3 Stmn2 Pfn2 Ywhah Apoe Prph1 Rab6b Calm3 Fstl1 Synpr Ubc Tubb3 Ywhag Pmp22 Pcp4 Ndn Tubb2a Serinc1 Dpysl3 Sncg Fabp7 Rgs4 S100a10 Stxbp1 :::::::: dorsal root ganglion pancreas trigeminal ganglion
26. Eef1a2 Pfn2 Ywhah Apoe Gpm6a Sparcl1 Ubc Ywhag Pcp4 Ndn Tubb2a Serinc1 Stxbp1 Zwint :::::::: amygdala cerebellum dorsal root ganglion hypothalamus olfactory bulb pancreas
27. Mgst1 Apoh Pgrmc1 Pon1 Vtn Cps1 Cat C3 Apoe Apoc4 Apoc1 Aldh2 Trf Ambp Hpxn Igfbp4 Aldh1l1 Ahsg Gc Tdo2 Atp5g3 Acaa2 Fabp1 Fgb Ubc Pah Plg F2 Pck1 Cyp2f2 Lamp1 Ubc Apoa2 Mat1a Gnmt Aldob Fbp1 :::::::: liver
28. Mgst1 Pgrmc1 Ehhadh Aadac Cat Gstt1 Apoe Aldh2 Atp5b Trf Sord Slc27a2 Igfbp4 Gc Atp5g3 Acaa2 Azgp1 Ubc Pah Mettl7b Alas1 Pck1 Crot Ubc Apoa2 Aldob Fbp1 Itm2b :::::::: kidney liver pancreas
29. Mgst1 Ghr Ehhadh Cat Gstt1 Apoe Aldh2 Fah Trf Igfbp4 Atp5g3 Acaa2 Ly6e Pah Pck1 Cox7b Itm2b :::::::: adipocyte kidney liver testis
30. Mgst1 Dcn Lgals1 Vim C3 Fabp4 Rps3 Col1a2 Ubc Pck1 Adipoq Rplp2 Cfd Cav1 Krt19 Dpt :::::::: adipocyte amygdala ovary trachea
31. Cpa1 Cpa2 Clps Serpini2 Clec4f Tff2 Ela1 Spink3 Ppib Pla2g1b Eef2 1810010M01Rik Clu Sycn Nupr1 P4hb Pnliprp1 Pnlip Cel :::::::: pancreas
32. Dctn6 Aebp1 Lama4 Srd5a2 Msh2 Zc3h13 Parp2 BC054438 Matn2 Fabp4 Serpine2 Bzw2 4930583H14Rik Narg2 Fnbp4 Prkar2b Tmem45b Osr2 Tmem100 Lamb2 Tmem43 Snx7 Ubtd2 G6pc Spon1 B3galnt1 Adipoq Cep57 Cfd Ndn F3 Grb10 Arl6 5730437N04Rik AB112350 Smoc2 Ktn1 Zmym1 Cmtm3 Stat1 Taf11 Ilf2 Prss35 Gpc3 Tie1 Pla2g5 Aspn Psat1 Emilin1 :::::::: adrenal gland liver ovary pancreas thyroid
33. 2410091C18Rik Myoc Pigh Agr2 Igfbp5 Zc3h13 Fshb Matn2 Tshb Serpine2 Bzw2 Crispld2 Gucy1a2 Angpt1 Tmem100 Hdgfrp3 Scg5 Tmem43 1810011O10Rik Gtpbp6 Pcp4 F3 Ktn1 Stt3a Ilf2 Acta1 Gpc3 Tacstd2 Galnt12 Tro Cenpb :::::::: liver ovary pituitary testis
34. Pdcd5 Pon1 Pon2 Ehhadh Reep6 Igfbp2 Cat C3 Gstt1 Apoe Lgals3 Calcrl Trf Ncor1 Scgb1a1 C1r Ndrg2 Tmem43 Alas1 Sftpc Cep57 Cyp2f2 Tubb2a Rnf13 Fbxl3 Tmem23 Oat Hprt1 Aox1 Itga8 Xdh :::::::: liver lung pancreas
35. Pdcd5 Pon2 Mgp Acadl Cd93 Jund1 Hrc Calcrl Ncor1 Cdh2 Scgb1a1 Ndrg2 Tmem100 Mfap4 Ppp1r14a Ndufa7 Tmem43 Myoz2 Alas1 Sftpc Cep57 Crip2 Slc36a2 Grb10 Fbxl3 Ktn1 Pln Mb Tmem23 Actn2 Hprt1 Xdh :::::::: heart lung pancreas
36. Pdcd5 Ehhadh Acadl Gstt1 Apoe Lgals3 Hexb Cyba Ncor1 Tmem45b Cmbl Tmem43 Alas1 Pck1 Cep57 Slc36a2 Arl6 2010310D06Rik BC038479 Tmem23 Hprt1 :::::::: adipocyte kidney pancreas
37. Gimap1 Aebp1 Ehhadh Srd5a2 Zc3h13 C3 Plvap BC054438 Matn2 Fabp4 Serpine2 Bzw2 Pnmt Snf1lk Aadacl1 Rom1 Tmem45b Gucy1a2 Flt1 Ict1 Scg2 Tmem43 Lrrn3 Cyfip1 1810011O10Rik F2r Pck1 Cfd Gimap6 Ndn F3 Ptn Fads1 Psmb10 Stat1 Gpc3 Tie1 Psmb9 Cyp21a1 Chgb Xdh Emilin1 :::::::: adrenal gland testis thyroid
38. Gimap1 Ehhadh Srd5a2 Zc3h13 Hrsp12 Matn2 Calcrl Snf1lk Tmem45b Tmem43 Snx7 Pck1 Pak2 Spock3 Tie1 Psmb9 Hemgn Xdh :::::::: adrenal gland pancreas testis
39. Gimap1 Ehhadh Myot Zc3h13 Snx9 Myh4 Wif1 Hoxb13 Slamf1 Snx7 Hrb Cep57 Arl6 Zmym1 Acta1 Bmi1 Serpinb5 :::::::: pancreas prostate thyroid
40. Gimap1 Ehhadh Zc3h13 Matn2 Serpine2 Calcrl Snf1lk Cd226 Tmem45b Tdo2 Slc6a1 Aqp3 Tmem100 Hdgfrp3 Slamf1 Tmem43 Snx7 Sema3e Tlr1 F3 Otx2 Spock3 S100a9 Crct1 Tie1 Synpo2l Hemgn :::::::: pancreas placenta testis thyroid
41. Gimap1 Srd5a2 Snx9 Calcrl Cdh2 Tmem45b D230037D09Rik Steap2 Snx7 Lrrn3 Hrb B3galnt1 Cep57 Arl6 2010310D06Rik Stat1 Tie1 Psmb9 Pla2g5 Bmi1 :::::::: adrenal gland pancreas thyroid
42. Afp Ehhadh 1700025G04Rik Prg1 C3 Lgals3 Matn2 Serpine2 Hexb Calcrl Nid1 Trf Tspan14 Lum Cstb Tmem100 Tmem43 Sema3e Tubb2a F3 Ktn1 Krt19 Acta1 Slc2a1 Gjb2 :::::::: pancreas placenta testis
43. Afp 1700025G04Rik Calcrl Rbm6 Tmem100 Ppp1r14a Snx7 Sema3e B3galnt1 Cep57 Tubb2a F3 Arl6 2010310D06Rik Otx2 Bzw1 Cyp17a1 Guca2b Synpo2l :::::::: pancreas placenta salivary gland
44. Afp Prg1 C3 Plvap Lgals3 Serpine2 Trf Ly6e Anxa5 Car4 F2r Krt19 :::::::: lung placenta testis
45. Slc45a4 Gimap1 Afp 1700025G04Rik Prg1 Snx9 Lgals3 Myh4 Serpine2 Cyba Trf Glipr1 Ncor1 Slc20a1 Grap Crsp9 Cstb Nudt9 9030416H16Rik Tmem43 Sema3e Hrb Cep57 Tubb2a Rasa3 Grb10 Bicd2 Efhc1 Zmym1 Rrm2 Tmem23 Acta1 S100a8 S100a9 Slc2a1 Hemgn Emilin1 :::::::: bone marrow pancreas placenta thyroid
46. Mgp Sparc C3 Gng11 Gstt1 BC054438 Fabp4 Xlkd1 Camk1 Pdgfrb Crispld2 Nid1 Flt1 Sparcl1 C1r Aqp5 Pcolce2 Tmem100 Mfap4 Car4 Sdpr Rnase4 Snx7 Spon1 1810011O10Rik Sftpc Cyp2f2 F3 Sox18 Cav1 Slc16a12 Plekhc1 Colec12 2900041A09Rik Krt19 Gpc3 Tacstd2 Tie1 zfp503 Aspn Itga8 :::::::: CD4+Tcells CD8+Tcells lung
47. Mgp Cat Gstt1 Plvap Acy3 Cyba Ly6e Cckar Car4 Dcxr Aqp1 1810011O10Rik Pck1 :::::::: kidney lung testis
48. Ghr Ehhadh Igfbp5 Cat Gstt1 Fabp4 Cyba Ly6e 1810011O10Rik Pck1 Cfd :::::::: adipocyte kidney testis
49. Ehhadh Srd5a2 1700025G04Rik Clpp Fshb Matn2 Tshb Serpine2 Mthfs Rasgrf1 Aga Arl6ip5 D230037D09Rik Nr2f6 Scg5 Ict1 Scg2 Tmem43 1810011O10Rik Pck1 Pcp4 Cyp2f2 Ndn Tubb2a Ptn Ktn1 Spock3 Psmb9 Chgb Xdh Esm1 :::::::: adrenal gland pancreas pituitary testis
50. Ehhadh Acadl Apoe Lgals3 Trf Prkar2b Ncor1 Tmem45b Ndrg2 Tmem43 Alas1 Pck1 Adipoq Cep57 Cfd Slc36a2 Grb10 2010310D06Rik Tmem23 Hprt1 Psmb9 :::::::: adipocyte adrenal gland pancreas
51. Ehhadh Igfbp5 Fabp4 Cd63 Egf Ly6e Spink3 Dcxr Pck1 Gjb2 :::::::: kidney prostate testis
52. Ehhadh Cat Fabp4 Etfa 1300013J15Rik Atp5g3 Ly6e Aqp1 1810011O10Rik Pck1 Acss1 Idh2 Cox7b :::::::: heart kidney testis
53. Dcn Reep1 Mt3 Eef1a2 Scg3 Stmn2 Aplp1 Rab3a NM_008963.2 Eno2 Sparc Igfbp5 Lrp11 Pfn2 N28178 Apoe Dusp26 Prph1 Avil Serpine2 Lxn Tagln3 Pla2g7 Fez1 Tmem45b Hspb8 Sparcl1 Rab6b 2700055K07Rik S100b Fstl1 Synpr Kcnab1 Tubb3 Snx7 9130213B05Rik Ywhag Mal Pmp22 Tac1 Lrrn1 Pcp4 Ndn Clstn2 Tubb2a Trim2 Plekhc1 Dpysl3 Apod Sncg Fabp7 Rgs4 Stxbp1 Chgb :::::::: CD4+Tcells CD8+Tcells dorsal root ganglion trigeminal ganglion
54. Dcn Ptpn18 Lgals1 Acp5 Agr2 Aebp1 Vim Mgp C3 Lgals3 Fabp4 Emp1 Cyba Crispld2 Cnn2 Nid1 Dbf4 Lum Nedd1 Rcn3 Myl9 Tmem45b C1r Col1a2 Osr2 Gas1 Mfap4 Lrg1 Dcxr G6pc Plac8 1810011O10Rik Pck1 Adipoq Cfd EG317677 1600029D21Rik Smoc2 Pigr Krt19 Acta1 Dpt Crabp2 Tacstd2 Cpt2 Psmb9 Aspn Emilin1 Heatr5a :::::::: amygdala hypothalamus olfactory bulb ovary trachea uterus
55. Dcn Esr1 Lama4 Prtn3 C3 Gstt1 Indo Matn2 Fabp4 Camk1 C530044N13Rik Rasgrf1 Pdgfrb Nid1 Fgfrl1 Wnt2 Tmem45b Cxcl13 C1r Nr2f6 Aqp5 Pcolce2 Cmbl Sdpr Rnase4 Snx7 Lrg1 Ssh3 G6pc Fzd4 1810011O10Rik Pck1 F3 Otx2 BC038479 Slc16a12 Colec12 Ptn 2900041A09Rik Krt19 Acta1 G0s2 Dpt Tacstd2 Tie1 zfp503 Aspn :::::::: CD4+Tcells CD8+Tcells adipocyte testis
56. Dcn Ccdc80 Lgals1 Acp5 Vim C3 Lgals3 Fabp4 Cyba Nid1 Lum Tmem45b Col1a2 Sdpr Lrg1 Slc1a5 G6pc 1810011O10Rik Pck1 Adipoq Cfd Cav1 Krt19 G0s2 Dpt Psmb9 :::::::: adipocyte amygdala
57. Dcn Krt23 Rab3d Agr2 Igfbp2 Igfbp5 Slc9a2 Ppp1r3c Myot C3 Gstt1 Foxa1 Hrc Krt15 Matn2 Myh4 Fabp4 Gchfr Crispld2 Nid1 Upk1b 2310045A20Rik Slc26a4 Hspb8 Sparcl1 Wif1 Hoxb13 C1r Pip Nr2f6 Azgp1 Aqp5 Krt4 Tmod4 Tgm4 Spink3 Slc5a8 Mfap4 Lrrc28 Sdpr Dcxr Apof 1810011O10Rik Mylpf Nucb2 Nupr1 Pcp4 2310057J18Rik Cyp2f2 Nr2f2 Slc16a12 Tmem30b Pigr Mb Krt19 Acta1 Efna1 Tacstd2 zfp503 Des Spdef Aspn Gjb2 :::::::: CD4+Tcells CD8+Tcells prostate
58. Dcn Lgals1 Rarres2 Afp Prg1 C3 Lgals3 Fabp4 Cd63 Serpine2 Trf Ly6e 1600029D21Rik Krt19 Gjb2 :::::::: ovary placenta testis
59. Dcn Mt4 Krt13 Krt15 Atp5b Rpl8 Rps3 Atp5a1 Atp5g3 Krt4 Ubc Rplp2 Mylpf Slpi Krtdap Mb Acta1 Crct1 Dsp :::::::: tongue trachea
60. Dcn Tfpi2 Rarres2 Afp Prg1 C3 Lgals3 Cd63 Serpine2 Trf Cstb Car4 Fgb Cdkn1c F2r Tfrc Krt19 Slc2a1 Guca2b Gjb2 :::::::: placenta testis
61. Dcn Rarres2 Tmem166 Igfbp2 C3 Matn2 Fabp4 Camk1 Serpine2 Pdgfrb Nid1 Hspb8 Flt1 Tmem100 Car4 Snx7 1810011O10Rik F3 Slc16a12 Colec12 Acvr2b Krt19 Gpc3 Guca2b Ghrh Apom Gjb2 :::::::: CD4+Tcells CD8+Tcells placenta
62. Dcn Mgp C3 Krt15 Fabp4 Ly6e Krt4 Pck1 Mylpf Cfd Cyp2f2 Mb Krt19 Acta1 :::::::: prostate testis trachea
63. Srd5a2 Id2 Apoe 4930583H14Rik Prkar2b Prdx3 Alas1 Pck1 Cfd Ndn Acly Cyp21a1 Chgb :::::::: adrenal gland pancreas
64. Tnp1 Prm2 Gstm5 Spata4 Ldhc Ubc Prm1 Tnp2 Tcp1 EG434402 Tcte3 Smcp :::::::: testis
65. D3Ucla1 C3 Aldh2 Atp5g3 Acaa2 Azgp1 Ly6e Herpud1 Ubc Eef2 Pck1 Cyp2f2 P4hb Lamp1 Ubc Pigr Gnmt Gjb2 :::::::: amygdala liver prostate salivary gland
