# Supplementary material for: Characterization and Comparison of the Tissue-Related Modules in Human and Mouse
Source: PLoS One. 2010 Jul 22;5(7):e11730. doi: 10.1371/journal.pone.0011730 (PMC2908688; doi:10.1371/journal.pone.0011730)
Supplement: Table S3 — Overlapped GO functional terms in five pairs of inter-species modules. (0.04 MB DOC) [file pone.0011730.s003.doc]

| Human module^a^ | | Mouse module | | Similarity | Overlapped GO terms |
| --- | --- | --- | --- | --- | --- |
| Genes (gene symbol) | Tissues | Genes (gene symbol) | Tissues |  |  |
| *APOH SERPIND1 RARRES2 VTN HRG REEP6 TTR C3 GLTSCR2 APOE APOC4 APOC1 COX4I1 APCS ALDH2 SLC27A5 TF AMBP HPX ITIH1 AHSG GC SERPING1 NNMT CD14 UBB DCXR AGXT EEF2 F2 CLU RPLP2 P4HB ACTB UBC CYP1A2 FXYD1 AGT APOA2 ECHS1 MAT1A FBP1* | liver | *Mgst1 Apoh Pgrmc1 Pon1 Vtn Cps1 Cat C3 Apoe Apoc4 Apoc1 Aldh2 Trf Ambp Hpx Igfbp4 Aldh1l1 Ahsg Gc Tdo2 Atp5g3 Acaa2 Fabp1 Fgb Ubb Pah Plg F2 Pck1 Cyp2f2 Lamp1 Uba52 Apoa2 Mat1a Gnmt Aldob Fbp1* | liver | 0.464^b^  0.451^c^ | acute inflammatory response  acylglycerol metabolic process  glycerolipid metabolic process  inflammatory response  lipid metabolic process  regulation of blood coagulation  regulation of coagulation  response to external stimulus |
| *EEF1A2 STMN2 APLP1 PTGDS SPARC TTR HSPA2 SYNGR3 APOE TUBB2B SYT1 TF FEZ1 GPM6A SPOCK1 SPARCL1 ZIC1 DDAH1 CHN1 GRIA2 NDRG2 SCG5 GPRC5B PMP22 CLU DKK3 PCP4 SV2B TUBB2A DNER APOD CKB CLIP3 STXBP1 CAMK2N1 CST3* | amygdala  cerebellum  hypothalamus | *Eef1a2 Pfn2 Ywhah Apoe Gpm6a Sparcl1 Ubb Ywhag Pcp4 Ndn Tubb2a Serinc1 Stxbp1 Zwint* | amygdala  cerebellum  hypothalamus  dorsal root ganglion  olfactory bulb | 0.351^b^  0.308^c^ | generation of neurons  neurogenesis |
|  |  | *Reep1 Mt3 Eef1a2 Scg3 Stmn2 Aplp1 Rab3a Ptgds Eno2 Sparc Igfbp5 Lrp11 Pfn2* *N28178 Apoe Dusp26 Prph1 Avil Serpine2 Lxn Tagln3 Pla2g7 Fez1 Tmem45b Hspb8 Sparcl1 Rab6b Tppp3 S100b Fstl1 Synpr Kcnab1 Tubb3 Snx7 9130213B05Rik Ywhag Mal Pmp22 Tac1 Lrrn1 Pcp4 Ndn Clstn2 Tubb2a Trim2 Fermt2 Dpysl3 Apod Sncg Fabp7 Rgs4 Stxbp1 Chgb* | dorsal root ganglion  trigeminal ganglion | 0.347^b^  0.291^c^ | anatomical structure development  multicellular organismal process  nervous system development  neurogenesis |
| *CKM MGP SPARC TNNC1 IGFBP5 C3 APOE APOC1 CSRP3 MYL9 HSPB8 MYL3 COX7A1 CLEC3B MFAP4 CD14 HSPB2 PMP22 C1QB CD151 CFD CLDN5 CAV1 PTRF TCF21 TIE1 MAFB* | heart  lung | *Hspb6 Pdk4 Pvalb Eef1a2 Mt4 Ckm Mgp Sparc Igfbp5 Myot Apobec2 C3 Krt13 Hrc Krt15 Myh4 Fabp4 Cd63 100039744 1110028A07Rik Rpl3l Hspb8 Sparcl1 Cox6a2 Cox7a1 Krt4 Tmod4 Cmbl Col1a2 Pgam2 Ndrg2 Sdpr Rnase4 Ckmt2 1810011O10Rik Mylpf Cfd Cyp2f2 Bgn Krtdap Pfkm Cav1 Fermt2 Ppl Thbs4 Fxyd1 Atp2a1 Myoz1 Acta1 Dpt Casq1 Crct1 Gsta4 Zfp503 Des Aspn Smpx* | skeletal muscle  tongue  trachea | 0.348^b^  0.224^c^ | regulation of muscle contraction |
| *MGP SPARC IGFBP5 C3 LUM MYL9 CLEC3B MFAP4 PMP22 C8orf4 CD151 CLDN5 KRT7 F3 CAV1 PPL WWTR1 KRT19 MAFB CLIC4* | lung | *Mgp Sparc C3 Gng11 Gstt1 Tmem204 Fabp4 Lyve1 Camk1 Pdgfrb Crispld2* *Nid1 Flt1 Sparcl1 C1r Aqp5 Pcolce2 Tmem100 Mfap4 Sdpr Rnase4 Snx7 Spon1 1810011O10Rik Sftpc Cyp2f2 F3 Sox18 Cav1 Slc16a12 Fermt2 Colec12 Tppp Krt19 Gpc3 Tacstd2 Tie1 Zfp503 Aspn Itga8* | lung | 0.297^b^  0.279^c^ | NA^d^ |

1. Note that the second human module has a relatively high similarity to the second and third modules of mouse in Table S3.
2. Shown is the modified similarity.
3. Shown is the similarity calculated by Eqs. (**1**).
4. We don’t observe overrepresented GO terms in the mouse module.
