# Supplementary figures and images for: Characterization and Comparison of the Tissue-Related Modules in Human and Mouse
Source: PLoS One. 2010 Jul 22;5(7):e11730. doi: 10.1371/journal.pone.0011730 (PMC2908688; doi:10.1371/journal.pone.0011730)

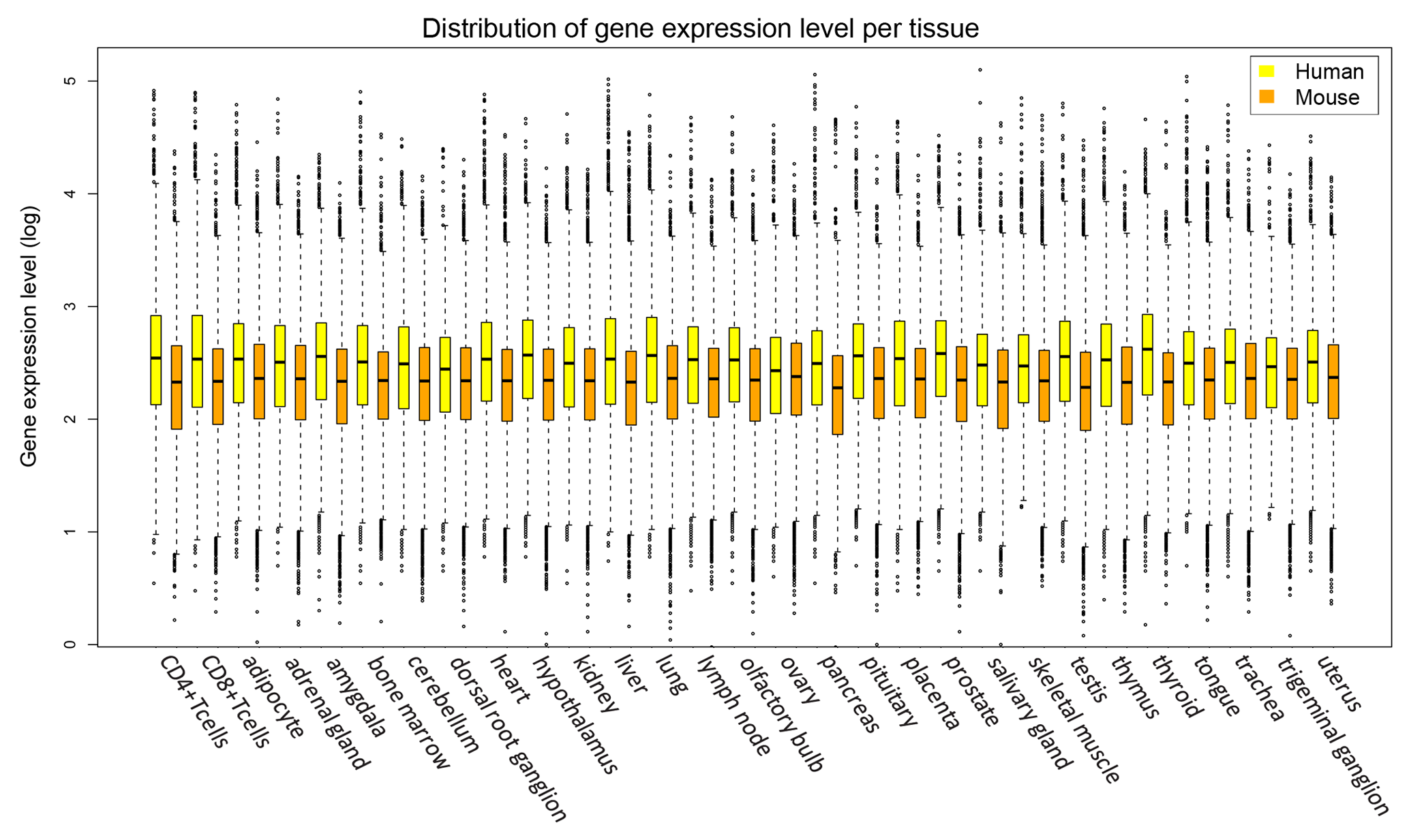

Supplement: Figure S1 — Gene expression pattern across tissues. The y-axis value is the logarithm of the gene expression level to the base 10. (1.54 MB TIF) [file pone.0011730.s004.tif]

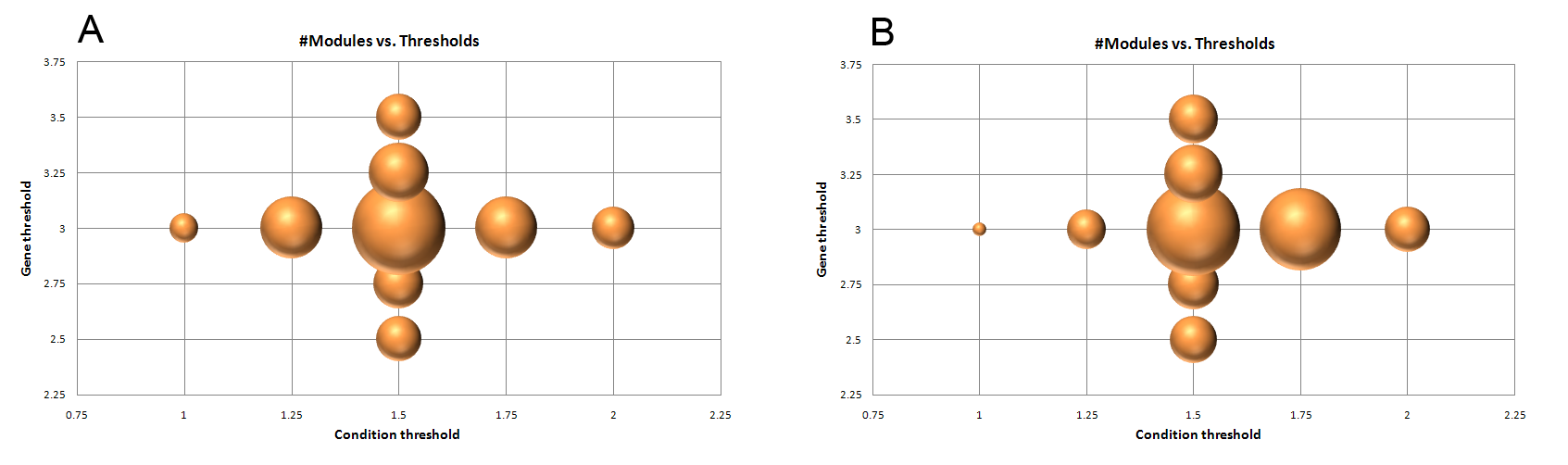

Supplement: Figure S2 — Relationship between the number of modules and the ISA thresholds used. (A) Human; (B) mouse. The number of modules is proportional to the area of the “Ball.” (0.14 MB TIF) [file pone.0011730.s005.tif]

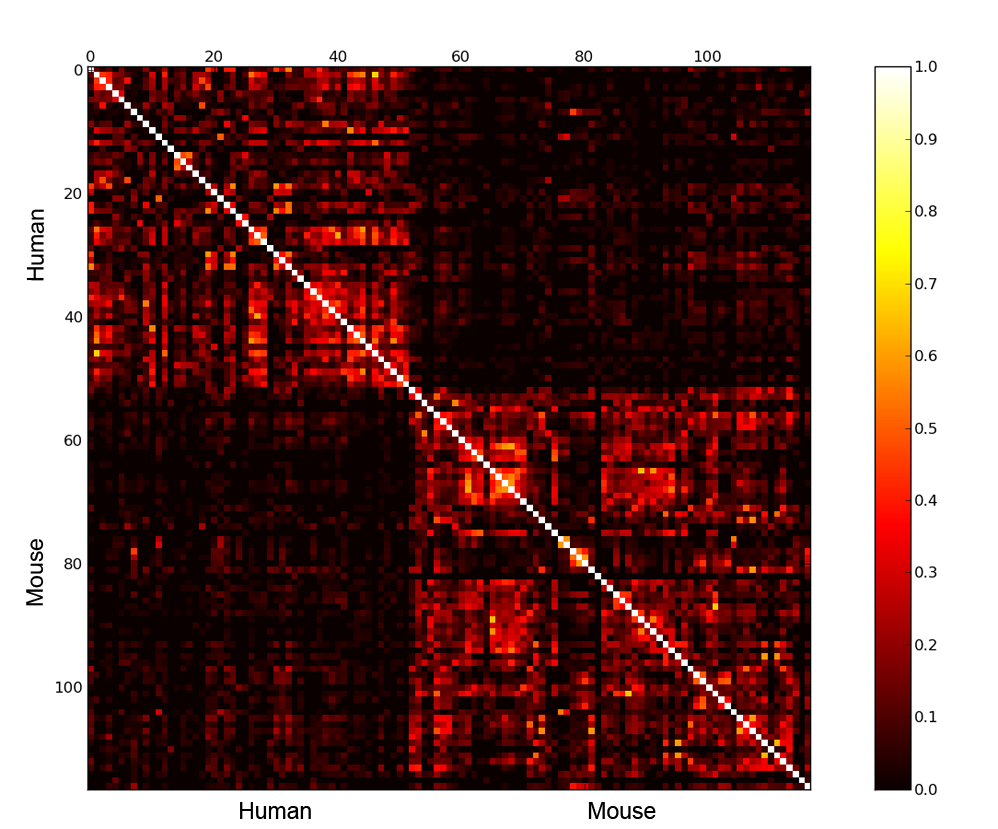

Supplement: Figure S3 — Similarity of modules within and between species. The heat map prominently displays a highly low similarity of modules from between species in contrast to those within each species. Rows and columns numbered 0–51 and 52–116 represent the human and mouse modules, respectively. (0.42 MB TIF) [file pone.0011730.s006.tif]

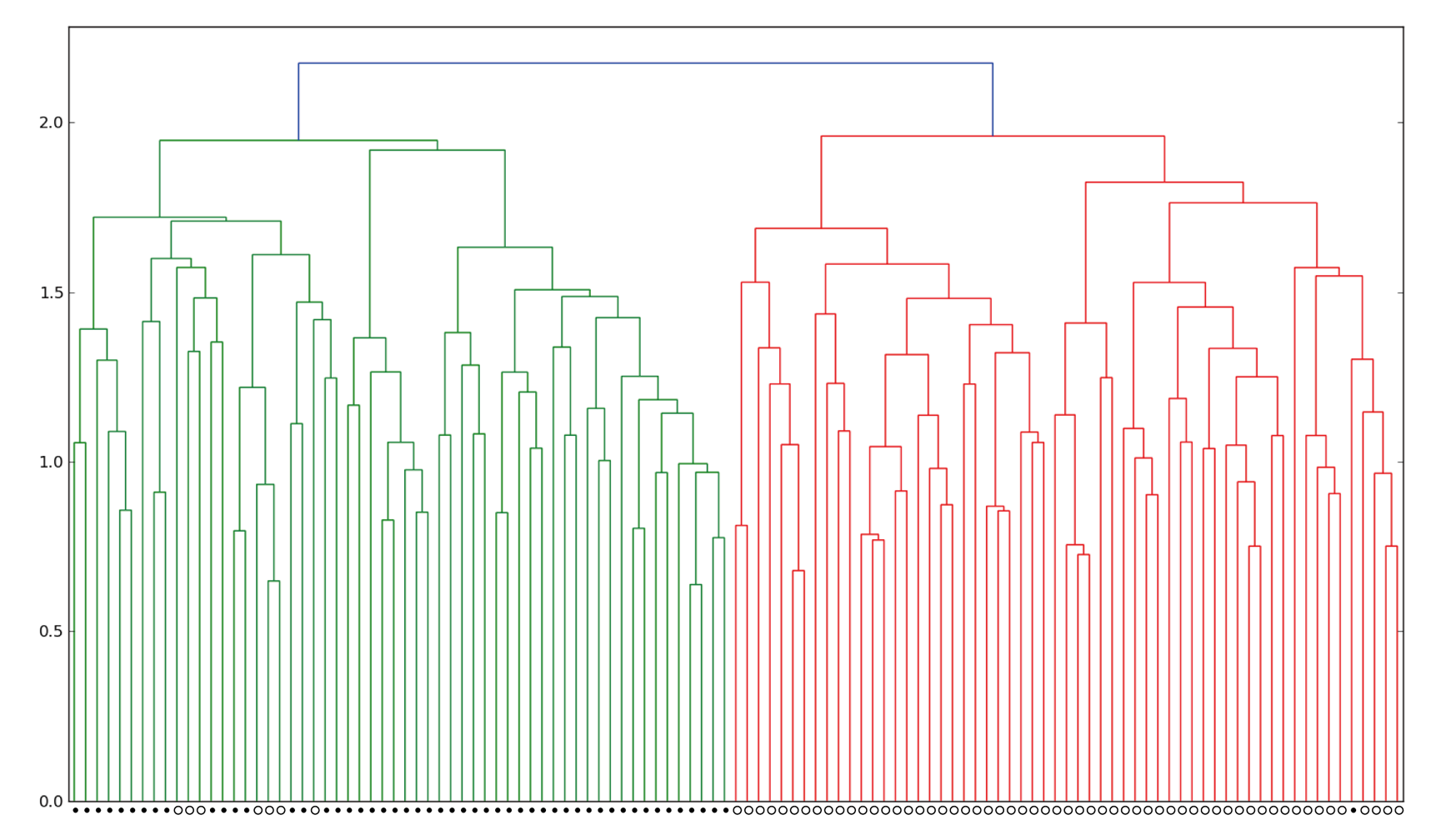

Supplement: Figure S4 — Hierarchical clustering graph of 117 (52 human and 65 mouse) modules. The tree indicates that only few pairs of the modules, which are derived from the two species respectively, have a relatively high overlap of genes. The filled cycles denote the human modules, and the unfilled cycles denote the human mouse modules. (0.87 MB TIF) [file pone.0011730.s007.tif]

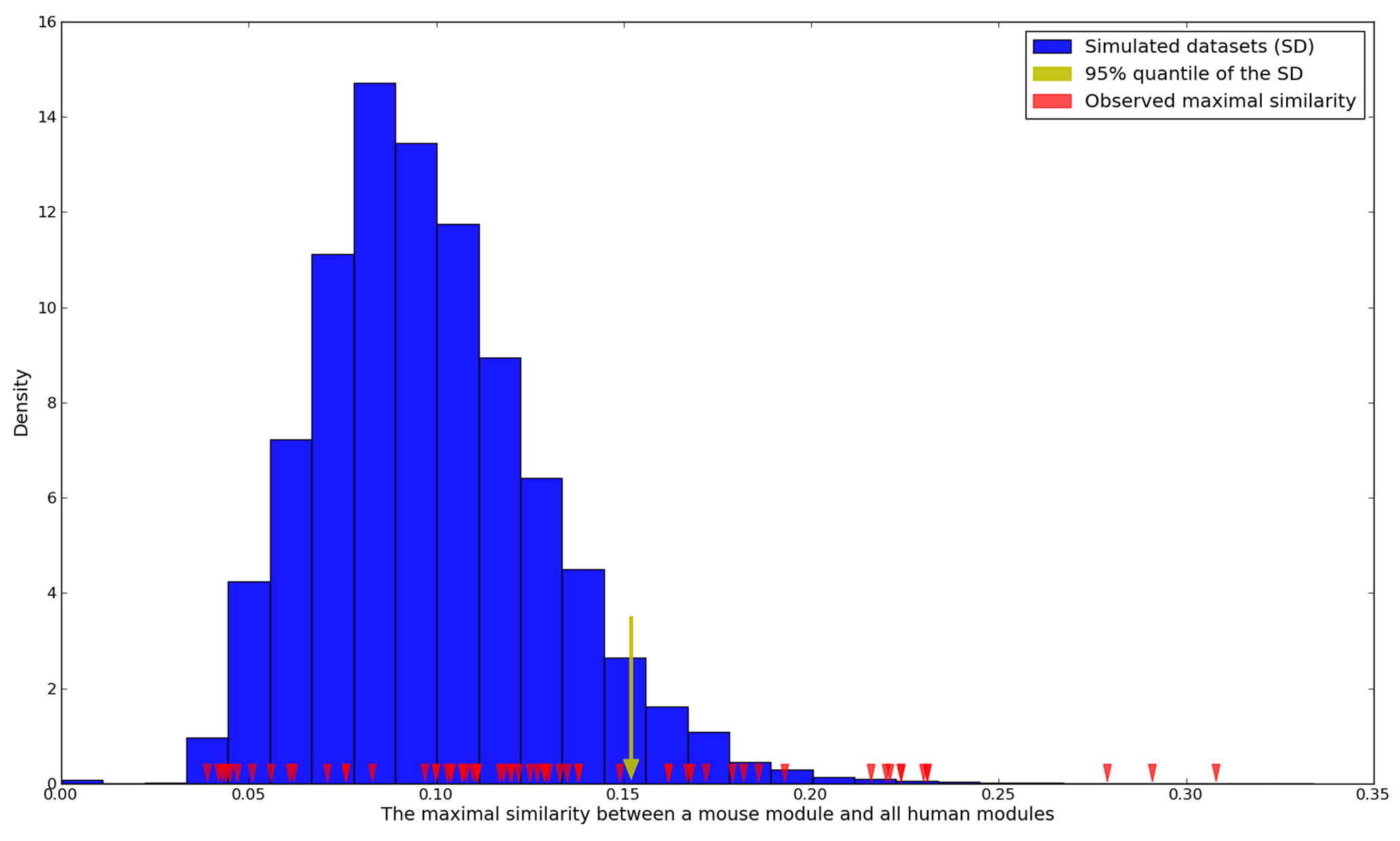

Supplement: Figure S5 — The statistical significance of the observed maximal similarity. The plot shows that a majarity of the interspecies modules have a low gene overlap. Note that a largest maximial simialrity (0.451) is not shown only for aesthetics. (0.47 MB TIF) [file pone.0011730.s008.tif]

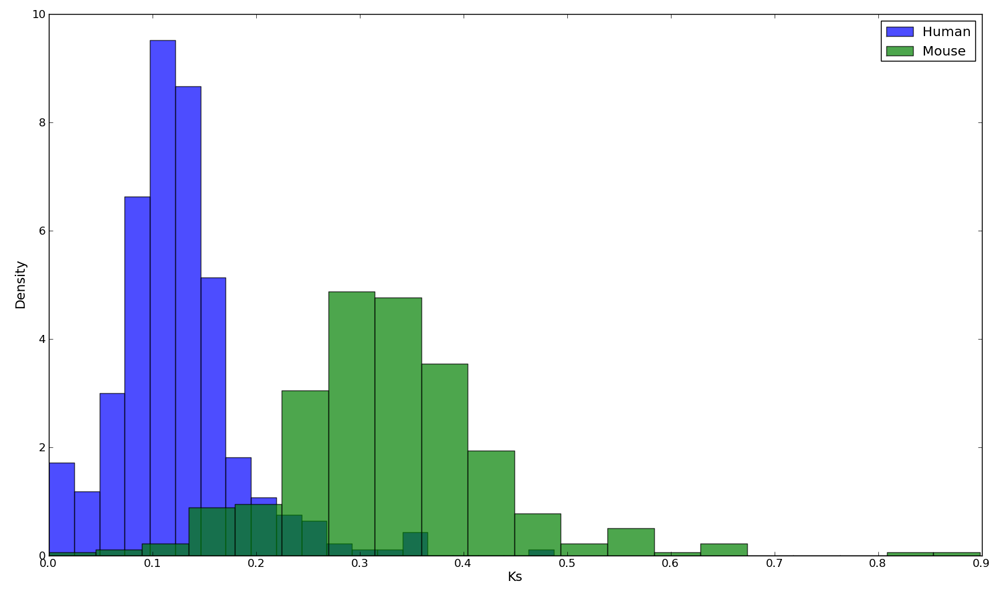

Supplement: Figure S6 — Histograph of the Ks in human or mouse lineage. (0.11 MB TIF) [file pone.0011730.s009.tif]
